# Systematic optimization of prime editing for enhanced efficiency and versatility in genome engineering across diverse cell types

**DOI:** 10.3389/fcell.2025.1589034

**Published:** 2025-04-29

**Authors:** Huiling Mu, Yeyi Liu, Yijia Chi, Fei Wang, Shuting Meng, Yi Zhang, Xunting Wang, Dongxin Zhao

**Affiliations:** ^1^ School of Chinese Materia Medica, Nanjing University of Chinese Medicine, Nanjing, China; ^2^ Shanghai Institute of Materia Medica, Chinese Academy of Sciences, Shanghai, China; ^3^ School of Pharmacy, University of Chinese Academy of Sciences, Beijing, China; ^4^ School of Pharmacy, Henan University, Kaifeng, China

**Keywords:** prime editing, piggyBac transposon system, pluripotent stem cells, sustained expression, genome engineering

## Abstract

Prime editing offers remarkable versatility in genome editing, but its efficiency remains a major bottleneck. While continuous optimization of the prime editing enzymes and guide RNAs (pegRNAs) has improved editing outcomes, the method of delivery also plays a crucial role in overall performance. To maximize prime editing efficiency, we implemented a series of systematic optimizations, achieving up to 80% editing efficiency across multiple loci and cell lines. Beyond integrating the latest advancements in prime editing, our approach combined stable genomic integration of prime editors via the piggyBac transposon system, selection of integrated single clones, the use of an enhanced promoter, and lentiviral delivery of pegRNAs, ensuring robust, ubiquitous, and sustained expression of both prime editors and pegRNAs. To further assess its efficacy in challenging cell types, we validated our optimized system in human pluripotent stem cells (hPSCs) in both primed and naïve states, achieving substantial editing efficiencies of up to 50%. Collectively, our optimized prime editing strategy provides a highly efficient and versatile framework for genome engineering *in vitro*, serving as a roadmap for refining prime editing technologies and expanding their applications in genetic research and therapeutic development.

## 1 Introduction

Prime editing represents a major breakthrough in gene editing, offering unprecedented precision and versatility in genetic modifications. Unlike the conventional CRISPR-Cas9 system (Gaj et al., 2013; Doudna and Charpentier, 2014; Sander and Joung, 2014; Ford et al., 2019), which primarily generates insertions and deletions (indels), or base editing (Komor et al., 2016; Gaudelli et al., 2017), which is restricted to specific types of nucleotide substitutions, prime editing enables precise insertions, deletions, and all 12 possible types of base substitutions without the need for an external donor template (Anzalone et al., 2019). By encoding the desired edit within a reverse transcription template, prime editing avoids double-strand DNA breaks, instead introducing a controlled single-strand incision, which significantly reduces the risk of unintended mutations and genomic instability (Anzalone et al., 2019). With its ability to generate a diverse range of genetic alterations while minimizing byproducts, prime editing has emerged as a powerful and efficient tool, broadening the scope of gene editing applications in both research and therapeutic settings.

Prime editing utilizes the SpCas9 H840A nickase (nCas9) fused with Moloney murine leukemia virus reverse transcriptase (M-MLV RT) to introduce precise genetic modifications based on an RNA template. This system employs a specialized prime editing guide RNA (pegRNA), an engineered single guide RNA (sgRNA) that includes an additional prime binding site (PBS) and a reverse transcription template encoding the desired edit at its 3′end (Anzalone et al., 2019). Under the guidance of the pegRNA, nCas9 nicks the DNA strand at the protospacer adjacent motif (PAM) site. The PBS sequence then anneals to the exposed 3′end of the cleaved DNA strand, initiating reverse transcription (Anzalone et al., 2019). Reverse transcriptase subsequently synthesizes a new DNA sequence based on the RT template, generating the intended genetic modification. Following this process, a dynamic equilibrium of 3′and 5′flap structures is formed at the nick site, which is then resolved through cellular repair mechanisms, ultimately incorporating the intended genetic modification (Anzalone et al., 2019).

Prime editing has shown great promise in genome editing due to its versatility and precision in genetic modifications. However, one of its major challenges is its relatively low editing efficiency, necessitating continuous optimization of the system. To address this, extensive efforts have been made to enhance prime editing performance, including structural and codon optimization of the nCas9-RT fusion enzyme (Chen et al., 2021), improvements in evading the mismatch repair pathway, and engineering more efficient pegRNAs (Nelson et al., 2021; Li et al., 2022b; Zhang et al., 2022). While these advancements have significantly boosted editing efficiency, much like the evolution of the CRISPR-Cas9 system, the effectiveness of prime editing is not solely determined by the editing machinery itself. Instead, it is also influenced by the expression level, stability, and duration of both the prime editor and pegRNAs, which are largely dictated by the delivery system. Therefore, optimizing delivery methods remains a critical factor in further improving prime editing efficiency.

The piggyBac (PB) transposon system is a DNA-based transposition system that facilitates gene transfer through a cut-and-paste mechanism. Due to its high efficiency in gene insertion, piggyBac has been widely utilized in genetic engineering and genome manipulation. PB is a 2,472 bp autonomous transposable element, flanked by 13 bp and 19 bp inverted terminal repeats (ITRs) at each end, and encodes a 594-amino acid transposase in its central region (Fraser et al., 1995; Fraser et al., 1996). The transposase encoded by the helper plasmid specifically recognizes and cleaves the inverted terminal repeats (ITRs) flanking the transposon plasmid, facilitating precise excision of the transposable element. This excised element is then integrated by the transposase into genomic TTAA tetranucleotide sites through a cut-and-paste mechanism (Fraser et al., 1996). The piggyBac transposon system exhibits substantial cargo capacity (20 kb) for multiplexed gene co-expression (Kahlig et al., 2010; Li et al., 2013), making it an optimal delivery platform for prime editor (PE) vectors. Furthermore, the piggyBac transposon system facilitates sustained transgene expression while circumventing the immunogenicity concerns associated with conventional viral delivery systems. To enhance prime editing efficiency, we employed the piggyBac transposon system for stable genomic integration and sustained expression of prime editor components.

In this study, we systematically evaluated the latest optimizations of prime editing machinery and explored strategies to enhance the exogenous expression of prime editors and pegRNAs. Our findings highlight an optimal approach for efficient prime editing, which is establishing single-cell clones with stable genomic integration of prime editors using the piggyBac transposon system, utilizing promoter for robust high-level gene expression of prime editor, and delivering epegRNAs via lentivirus to ensure sustained expression for up to 14 days. By combining improvements in both editing efficiency and delivery methods, we achieved up to 80% editing efficiency across multiple cell lines and genomic loci. Furthermore, we validated our approach in more challenging cell types, including human pluripotent stem cells in both primed and naïve states, achieving more than 50% editing efficiency.

## 2 Materials and methods

### 2.1 Plasmid construction

The plasmids pCMV-PE2 (Addgene, #132775), pCMV-PEmax-P2A-hMLH1dn (Addgene, #174828), and pB-CAGGS-dCas9-KRAB-MeCP2 (Addgene, #110824) were all obtained from Addgene. To construct the pB-pCMV-PEmax-P2A-hMLH1dn vector for piggyBac DNA transposition, we first digested the pCMV-PEmax-P2A-hMLH1dn plasmid with SpeI and PmeI to obtain a 9,390 bp fragment. We then digested the pB-CAGGS-dCas9-KRAB-MeCP2 plasmid with SpeI and PmeI to obtain a 5,772 bp backbone fragment. Finally, the two fragments were ligated using T4 DNA ligase (NEB, M0202S).

The pB-pCMV-PEmax-P2A-hMLH1dn vector was further modified by replacing the CMV promoter with the CAG promoter, resulting in the pB-pCAG-PEmax-P2A-hMLH1dn vector. The pB-pCMV-PEmax-P2A-hMLH1dn vector was digested with SpeI and SalI. The CAG promoter (1,741 bp) was amplified from the pCAG-hyPBase plasmid using pCAG-F and CAG-R primers, and T7-SV40 NLS-Cas9 (141 bp) was amplified from the pCMV-PEmax-P2A-hMLH1dn plasmid using T7-cas9-F and SV40NLS-Cas9-R primers. The digested pCMV-PEmax-P2A-hMLH1dn vector (14,465 bp) and two PCR fragments were then assembled using the 2 × MultiF Seamless Assembly Mix (ABclonal Technology, Wuhan, China) to generate the pB-pCAG-PEmax-P2A-hMLH1dn vector.

To facilitate the establishment of piggyBac prime editing cell lines, a T2A-mCherry fragment was subsequently inserted downstream of the MLH1 del754-756 codon. The pB-pCAG-PEmax-P2A-hMLH1dn plasmid was digested with SbfI and PmeI. Simultaneously, the hMLH1dn fragment (761 bp) was amplified from the pB-pCAG-PEmax-P2A-hMLH1dn plasmid using MLH1dn-SbfI-F and MLH1dn-R primers, and the T2A-mCherry fragment (828 bp) was amplified from the Lenti-dCas9-Zim3-mCherry plasmid (lab-generated) using T2A-mCherry-F and mCherry-PmeI-R primers. The linearized vector (15,502 bp) and two PCR fragments were then assembled using the 2 × MultiF Seamless Assembly Mix to generate the pB-pCAG-PEmax-P2A-hMLH1dn-T2A-mCherry vector.

To construct the pCAG-hyPBase plasmid, we synthesized the AgeI-KOZAK-hyPBase-NotI template using gBlock. The hyPBase fragment (1,857 bp) was amplified using pCAG-AgeI-Kozak-hyPBase-F and hyPBase-NotI-pCAG-R primers with AgeI-KOZAK-hyPBase-NotI as the template. The CAG-CBE4max-SpG-P2A-EGFP plasmid (Addgene #139998) was digested with AgeI-HF and NotI-HF, and the digested CAG-CBE4max-SpG-P2A-EGFP vector (4,826 bp) and hyPBase fragment were then assembled using Gibson Assembly.

To generate the epegRNA vector, we first constructed the Lenti-TevopreQ1-Puro backbone starting from the Lenti-guide-puro vector (Addgene #52963) for epeg guide cloning. The Lenti-guide-puro vector was digested with NsiI-HF and XmaI to remove the original sgRNA scaffold region. The insert regions were then PCR-amplified to introduce the TevopreQ1 motif. The Lenti-guide-puro plasmid was used as a template to amplify the region 1 fragment with primers Align-LGP-NsiI-F and LGP-BsmBI- tevopreQ1-R, and the region 2 fragment with primers tevopreQ1-T6-LGP-align-F and Align-LGP-XmaI-R. Finally, the digested vector (9,746 bp), region 1 fragment (202 bp), and region 2 fragment (293 bp) were assembled using Gibson Assembly.

The epeg guides were cloned into the Lenti-TevopreQ1-Puro vector to generate the optimized epegRNA. The Lenti-TevopreQ1-Puro vector was digested using BsmBI. The F + E scaffold fragment (86 bp) was generated by PCR reaction, and the epeg guide cloning insert was amplified using the F + E scaffold fragment as a template. Finally, the epeg guide cloning insert was assembled into the digested Lenti-TevopreQ1-Puro vector (8,259 bp). All PCR primers are listed in Supplementary Table S1, and the plasmid construction system is described in the Supplementary Material.

### 2.2 Cell culture and transfection

293T (CRL-3216) and HeLa (CRM-CCL-2) cells were purchased from the American Type Culture Collection (ATCC). MCF7 and T47D cells were acquired from Xiaohua Chen Lab. H1 human embryonic stem cells were acquired from WiCell. 293T and MCF7 cells were cultured in Dulbecco’s Modified Eagle Medium (DMEM, Meilunbio, Dalian, China) supplemented with 10% fetal bovine serum (FBS) and 1% Penicillin/Streptomycin (Pen/Strep). T47D cells were cultured in RPMI-1640 medium (Meilunbio, Dalian, China) with 10% FBS and 1% Pen/Strep. HeLa cells were cultured in Minimum Essential Medium (MEM, Meilunbio, Dalian, China) with 10% FBS and 1% Pen/Strep. Pluripotent stem cells were cultured in Pluripotency Growth Master 1 (CA1007500, CellApy Biotechnology) and the medium was changed every other day. All cell lines were maintained with 5% CO2 at 37°C. Cells were seeded in 6-well plates before transfection. Transfection was performed at 80% confluency using 12 μL of Lipofectamine 2000 (Invitrogen, Thermo Fisher Scientific) with 2 μg of PE and 2 μg of pegRNA, following the manufacturer’s instructions. For lentiviral infection of pegRNA, 400 μL of lentivirus and a final concentration of 8 μg/mL of polybrene (Sigma-Aldrich, Catalog No. 638133) were added to the cells. To establish the piggyBac prime editing cell lines, cells were transfected with 2 μg of pB-pCAG-PEmax-P2A-hMLH1dn-T2A-mCherry and 2 μg of pCAG-hyPBase using Lipofectamine 2000. Blasticidin selection (10 μg/mL, Beyotime, Shanghai, China) was initiated 24 h post-transfection, with medium replacement every 3 days. The antibiotic selection was maintained for 3 weeks. Next, mCherry-positive PB-PE single clones were identified by fluorescence microscopy and isolated using 10 μL pipette tips for expansion in 12-well plates.

### 2.3 Lentivirus production

293T cells were seeded into 10 cm dishes and transfected when the cell density reached 70%. Add 20 μg of plasmid DNA (2.5 μg of pMD2.G, 10 μg of psPAX2, and 7.5 μg of lentiviral vector) into a centrifuge tube containing Opti-MEM (Gibco, Thermo Fisher Scientific), bringing the final volume to 500 μL, and mix well. Then, add 50 μL of PEI transfection reagent to a separate centrifuge tube containing 450 μL of Opti-MEM, bringing the final volume to 500 μL, and mix well. Next, add the 500 μL of PEI transfection reagent solution dropwise to the plasmid solution, mix gently, and incubate at room temperature for 15 min. Finally, add 1 mL of the PEI/plasmid complex dropwise and evenly to the cell culture dish. Twelve hours after transfection, the medium was replaced with fresh complete medium. Forty-eight hours post-transfection, the culture supernatant was collected into a 50 mL centrifuge tube and stored temporarily at 4°C. Fresh complete medium was added to the cell culture dish to continue culturing. Seventy-two hours post-transfection, the supernatant was collected again and combined with the previous collection. The mixture was then centrifuged at 1500 rpm for 5 min to remove cells and cell debris. The virus supernatant was filtered using a 0.45 μm filter and stored at −80°C.

### 2.4 Genomic DNA extraction and PCR

Genomic DNA was extracted from the collected cells using the Universal Genomic DNA Kit (CWBIO, Shanghai, China). DNA amplification was performed using 2 × KAPA HiFi Hot Start Polymerase (KAPA Biosystems) unless otherwise specified. The primers used for genomic amplification are listed in Table 1. The PCR program was set as follows: initial denaturation at 95°C for 3 min, followed by 28 cycles of denaturation at 98°C for 20 s, annealing at 60°C for 15 s, and extension at 72°C for 30 s. A final extension was performed at 72°C for 5 min, and the reaction was held at 4°C. The PCR products were purified using 1.2 × VAHTS DNA Clean Beads (Vazyme, Nanjing, China), and then subjected to a second-round PCR to add Illumina adapters and index sequences. The second-round PCR program was: 95°C for 3 min, followed by 6 cycles of (98°C for 20 s, 65°C for 15 s, 72°C for 30 s), with a final extension at 72°C for 5 min, and storage at 4°C. The second-round PCR products were purified again using 1.2 × VAHTS DNA Clean Beads, quantified with a Qubit fluorometer, and subsequently subjected to NovaSeq paired-end sequencing.

**TABLE 1:** Primer for deep sequencing.

Target/Direction	Sequence (5′-3′)
HEK3-Fwd	ACACTCTTTCCCTACACGACGCTCTTCCGATCTNNNNATGTGGGCTGCCTA GAAAGG
HEK3-Rev	GACTGGAGTTCAGACGTGTGCTCTTCCGATCTCCCAGCCAAACTTGTCAACC
RNF2-Fwd	ACACTCTTTCCCTACACGACGCTCTTCCGATCTNNNNACGTCTCAT ATGCCCCTTGG
RNF2-Rev	GACTGGAGTTCAGACGTGTGCTCTTCCGATCTACGTAGGAATTTTGGTGGGACA
CDK4-Fwd	ACACTCTTTCCCTACACGACGCTCTTCCGATCTNNNNTGCTACGGGCAATCAC TCTC
CDK4-Rev	GACTGGAGTTCAGACGTGTGCTCTTCCGATCTCCGAGATCTGAAGCCAGGAACA
RB1-Fwd	ACACTCTTTCCCTACACGACGCTCTTCCGATCT NNNNCGAACACCCAGGCGAGGTCA
RB1-Rev	GACTGGAGTTCAGACGTGTGCTCTTCCGATCTAGAAGTAAATATTGTTAGGGAG
AAVS1-Fwd	ACACTCTTTCCCTACACGACGCTCTTCCGATCTNNNNGCAGGG CAGGGAAGGAGACA
AAVS1-Rev	GACTGGAGTTCAGACGTGTGCTCTTCCGATCTCTGGCTTTGGCAGCCTGTGCTG
oct4 K177A-Fwd	ACACTCTTTCCCTACACGACGCTCTTCCGATCTNNNNTCCCTGAACCTAGTGGG GAG
oct4 K177A-Rev	GACTGGAGTTCAGACGTGTGCTCTTCCGATCTCTTCCTCCACCCACTTCTGC

### 2.5 RT-qPCR

293T cells were seeded in 6-well plates before transfection. Transfection was performed at 80% confluency using 12 μL of Lipofectamine 2000 (Invitrogen, Thermo Fisher Scientific) with 2 μg of PE4max. Meanwhile, 400 μL of pre-packaged pegRNA or epegRNA lentivirus was added to the culture medium, supplemented with 8 μg/mL polybrene (Sigma-Aldrich, Catalog No. 638133) to facilitate viral entry. After 7 days, total RNA was extracted using the Super FastPure Cell RNA Isolation Kit (Vazyme, Nanjing, China). Reverse transcription was performed according to the steps of the Reverse Transcriptase (HeavyBio, Wuhan, China) to obtain cDNA. Quantitative PCR analysis was performed on the scaffold regions of both pegRNA and epegRNA transcripts using target-specific primers, following the protocol of the Taq Pro Universal SYBR qPCR Master Mix (Vazyme, Nanjing, China). Primer sequences are provided in Supplementary Material. The thermodynamic program was performed under the following conditions: 95°C for 3 min, followed by 35 cycles of (98°C for 20 s, 55°C for 15 s, 72°C for 20 s). The relative expression levels of pegRNA and epegRNA were analyzed by comparing their 2^−ΔΔCq^ values.

### 2.6 Statistical analysis

Statistical analyses were carried out using GraphPad Prism nine software, unless otherwise noted. The corresponding analyses are described in the figure legends. Data are represented as the mean ± SD of three or four different experiments. P values were obtained using the 2-way ANOVA with Bonferroni multiple comparisons test. p < 0.05 indicated a statistically significant difference in the results.

## 3 Results

### 3.1 Benchmarking the optimization of prime editing machinery

**FIGURE 1:**
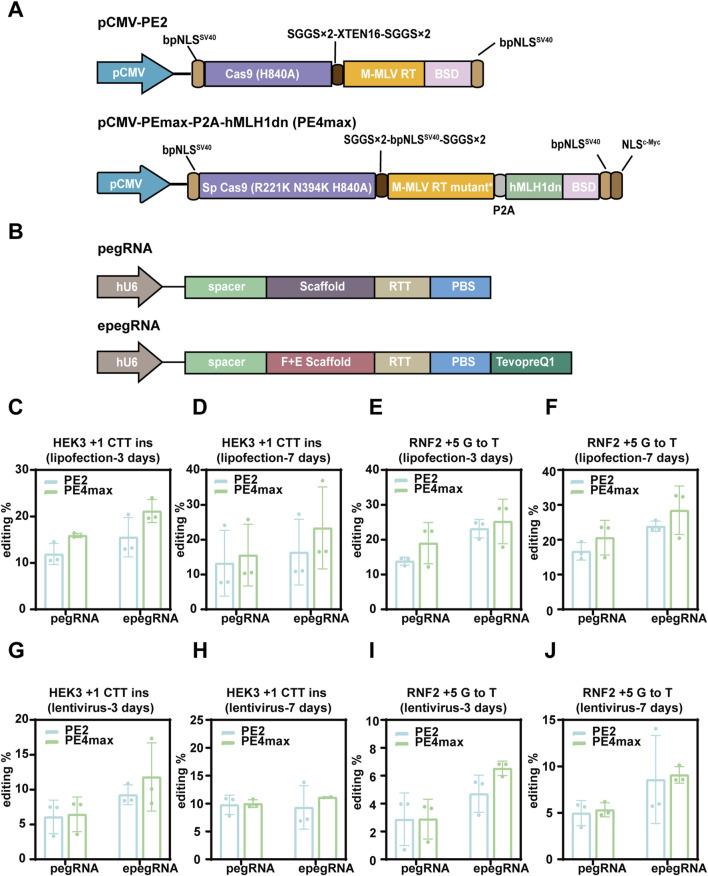
The impact of benchmark optimization on the efficiency of prime editing. **(A)** Schematic representation of the PE2 system and the codon-optimized PE4max system. **(B)** Schematic representation of the pegRNA and engineered epegRNA. **(C–J)** Comparison of the effects of various delivery methods, editing durations, and pegRNA modifications on prime editing efficiency in the PE2 and PE4max systems. All PE2 and PE4max systems were transiently transfected into 293T cells, while the corresponding pegRNAs were delivered via lipofectamine and lentiviral methods, respectively. The editing durations were 3 days and 7 days, respectively. **(C–F)** Demonstrate the editing efficiency of HEK3 and RNF2 loci when transiently transfecting pegRNAs. **(G–J)** The editing efficiency at the HEK3 and RNF2 loci with lentiviral delivery of pegRNAs. The results are represented as mean ± SD (n = 3).

Prime editing relies on two key components: an engineered enzyme, which is a fusion of reverse transcriptase and Cas9 nickase, and a prime editing guide RNA (pegRNA), which includes a spacer sequence to direct the Cas9 fusion protein to the target site and a 3′extension template encoding the desired genetic modification (Anzalone et al., 2019). To enhance the efficiency of prime editing beyond the initial version (PE2), previous studies have introduced several advancements. These include modifications to the mismatch repair (MMR) pathway through co-expression of dominant negative MLH1 (hMLH1dn) in PE4, optimization of the prime editor codon and architecture in PEmax (Chen et al., 2021), and the engineering of enhanced pegRNAs (epegRNAs) to preserve pegRNA stability and integrity (Nelson et al., 2021). To evaluate these advancements, we compared the performance of the PE2 and PE4max systems by lipofectamine-mediated co-transfecting 293T cells with plasmids encoding pegRNAs and prime editors. Among them, PE4max was an optimized framework based on PE2. Specifically, MLH1dn (a DNA mismatch repair repressor) was co-expressed with PE2 to create the PE4 system (PE2 + MLH1dn). Furthermore, in PE4max, SpCas9 (H840A) from PE4 was replaced with the SpCas9 (R221K, N394K, H840A) variant, and a human codon-optimized reverse transcriptase (RT) was used (Chen et al., 2021). Additionally, a 34-aa linker containing a bipartite SV40 NLS and an extra C-terminal c-Myc NLS was incorporated to enhance functionality (Chen et al., 2021), resulting in the PE4max system. The PE2 and PE4max editors, driven by the CMV promoter, were cloned into plasmids for transient expression (pCMV-PE2 for PE2 and pCMV-PEmax-P2A-hMLH1dn for PE4max, Figure 1A).

We also compared the original pegRNA architecture with the engineered epegRNA design, which includes an optimized scaffold and an RNA stabilization motif, tevopreQ1, positioned downstream of the primer-binding site (Figure 1B). The original sgRNA scaffold was modified to generate an optimized ‘flip and extension’ (F + E) sgRNA scaffold. This modification included a U-A to A-U substitution at the fourth base pair to prevent a potential Pol III terminator from affecting pegRNA expression, as well as a 5 bp extension in the hairpin structure to enhance Cas9 binding (Chen et al., 2013; Nelson et al., 2021). Prime editing efficiency was evaluated for a +1 CTT insertion at the HEK3 locus and a +5 G-to-T transversion at the RNF2 locus. Next-generation sequencing (NGS) revealed that the intended edits were successfully installed as early as 3 days post-transfection, with editing efficiencies reaching 23.37% and 28.45% by day 7 at the HEK3 and RNF2 loci, respectively (Figures 1C–F). Simultaneously, we quantified pegRNA and epegRNA transcripts via RT-qPCR in the PE4max system at 7 days post-lentiviral infection. Quantitative analysis revealed that epegRNA transcript levels were significantly higher than those of pegRNA at both the HEK3 and RNF2 target loci (Supplementary Figure S3A–B). Consistent with previous reports, the epegRNAs outperformed the original pegRNA designs, underscoring the advantages of RNA stabilization motifs in the epegRNA architecture (Figures 1C–F).

Despite utilizing the high-performing PE4max system and optimized epegRNAs, the intended editing efficiency remains constrained with lipofection-based delivery of the prime editing machinery, suggesting the need for improved delivery strategies. Viral delivery systems, such as lentivirus, are among the most commonly used and efficient modalities. Although lentivirus has a packaging capacity of up to 10 kb, which is insufficient for the PE4max expression cassette. To address this, we delivered the pegRNAs via lentivirus while continuing to use lipofectamine for the PE4max editor. However, this approach showed no better or even worse editing efficiency compared to delivering both pegRNAs and the prime editor using lipofectamine (Figures 1G–J), likely due to the lower expression levels achieved with lentivirus. These findings suggest that alternative strategies for the expression of prime editing machinery are crucial for optimizing prime editing efficiency.

### 3.2 Stable expression of prime editor cassette and epegRNAs for achieving efficient prime editing

**FIGURE 2:**
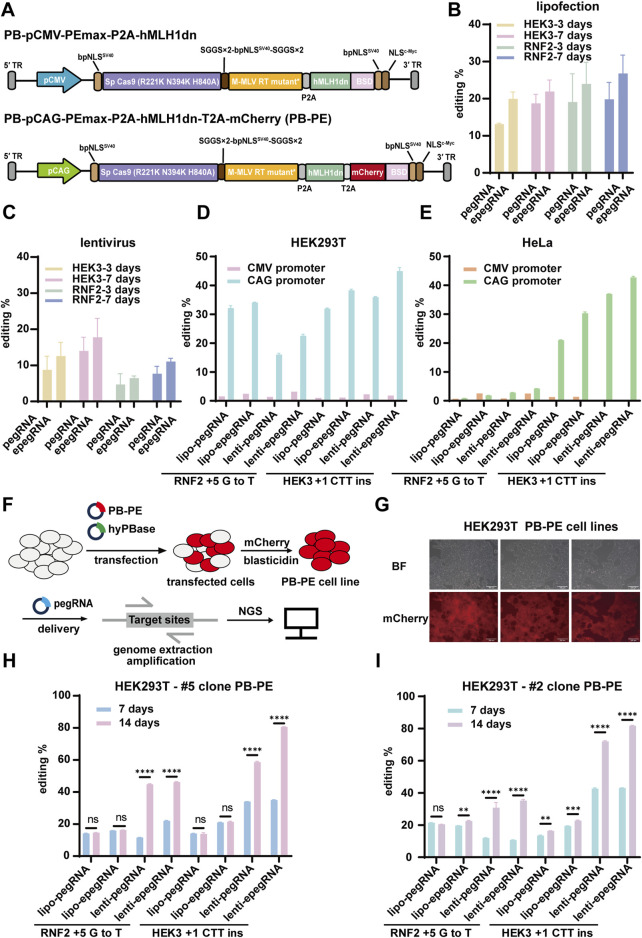
Prime editing using the PB-PE system in 293T cells. **(A)** Schematic representation of PB-PE utilized for piggyBac transposition. PB-pCMV-PEmax-P2A-hMLH1dn is the original PB-PE system, while PB-pCMV-PEmax-P2A-hMLH1dn is the optimized version, featuring promoter enhancement and mCherry reporter fusion. **(B,C)** The working performance was validated through liposomal delivery of PB-PE. The editing periods for the RNF2 and HEK3 loci were 3 and 7 days, respectively, with pegRNAs delivered via lipofectamine and lentivirus. PegRNAs delivered via lipofectamine are shown in **(B)**, and pegRNAs delivered via lentivirus are shown in **(C)**. **(D)** In 293T cells, the substitution of CMV and CAG promoters affects editing efficiency. **(E)** Impact of different promoters on the editing efficiency of the PB-PE system in HeLa cells. **(F)** Procedure for establishing PB-PE integrated cell lines and performing prime editing. **(G)** Integrated 293T PB-PE cell lines expressing mCherry fluorescence; scale bar, 200 µm. **(H,I)** The editing efficiency of the two single clones was evaluated at both 7 days and 14 days post-delivery. Data are represented as mean ± SD (n = 3). P values were obtained using the 2-way ANOVA with Bonferroni multiple comparisons test. ** p < 0.01; *** p < 0.001; **** p < 0.0001; ns, p > 0.05.

Next, we investigated the strategy of stably integrating the prime editor cassette into the genome of mammalian cells using piggyBac-based DNA transposition, leveraging its large integration capacity. First, we cloned the prime editor enzyme and hMLH1dn, both driven by the CMV promoter, into a piggyBac vector. We confirmed that this vector functioned effectively as a transient expression system when delivered via lipofectamine (Figures 2A–C). We then co-transfected this payload plasmid with a hyperactive piggyBac transposase (hyBase) to facilitate genomic integration and generated stable 293T cell lines expressing the prime editor. Unexpectedly, these stable cell lines exhibited significantly lower editing efficiency compared to their counterparts expressing the same cassette through lipofection (Figure 2D). We repeated this experiment in HeLa cells and observed the same trend (Figure 2E), indicating the need for further optimization of the piggyBac system.

We speculate that this low editing efficiency may be due to silencing and decreased expression of the CMV promoter over time following genomic integration. To address this issue, we replaced the CMV promoter with the CAG promoter, a synthetic strong promoter that combines the cytomegalovirus enhancer with the chicken β-actin promoter and first intron. Additionally, we incorporated an mCherry reporter gene linked via a 2A peptide to monitor the expression of the prime editor and hMLH1dn. These modifications led to the development of an optimized piggyBac system for prime editing (Figure 2A) and the generation of stable piggyBac prime editing cell lines through hyPBase-mediated genomic integration (Figures 2F,G). Compared to the previous piggyBac cells with the CMV promoter, the new piggyBac cells with the CAG promoter exhibited an average 17.29-fold increase in editing efficiency (Figure 2D). In 293T pooled cells, the editing efficiencies for HEK3 +1 CTT ins and RNF2 +5 G to T reached up to 44.98% and 34.09%, respectively (Figure 2D). These results suggested that the CAG promoter is crucial for efficient prime editing using the piggyBac transposon system.

To ensure stability and efficiency, we selected two single clones (#2 and #5) from the pooled cells with integrated prime editor, as piggyBac integration occurs randomly across the genome with certain biases. We then delivered pegRNAs using either lipofectamine or lentivirus and evaluated editing efficiency at both 7 days and 14 days post-delivery. Notably, with lentiviral transduction, editing efficiency continued to increase up to 14 days, suggesting that extending the editing duration can enhance efficiency (Figures 2H,I). However, this trend was not observed with lipofectamine-based delivery, likely due to the rapid degradation and dilution of transiently expressed pegRNAs as cells proliferate. The highest editing rates at the HEK3 locus reached 81.78% and 80.62% for clones #2 and #5, respectively, when epegRNAs were delivered via lentivirus and assessed at 14 days. Compared to pegRNAs targeting the HEK3 locus, those targeting the RNF2 locus were less efficient, achieving editing rates of up to 35.22% and 46.22% for clones #2 and #5, respectively (Figures 2H,I).

In conclusion, we achieved prime editing efficiency exceeding 80% through several key optimizations. First, we utilized the piggyBac transposon system for stable integration of the prime editing machinery into the genome. Second, we employed the CAG promoter to ensure sustained expression of the prime editor enzymes. Third, isolating single clones using a fluorescent reporter provided stable and consistent results. Lastly, we delivered epegRNAs via lentivirus to extend the editing duration effectively. Together, these optimizations led to a substantial improvement in editing efficiency.

### 3.3 Efficient prime editing across multiple loci and cell lines

**FIGURE 3:**
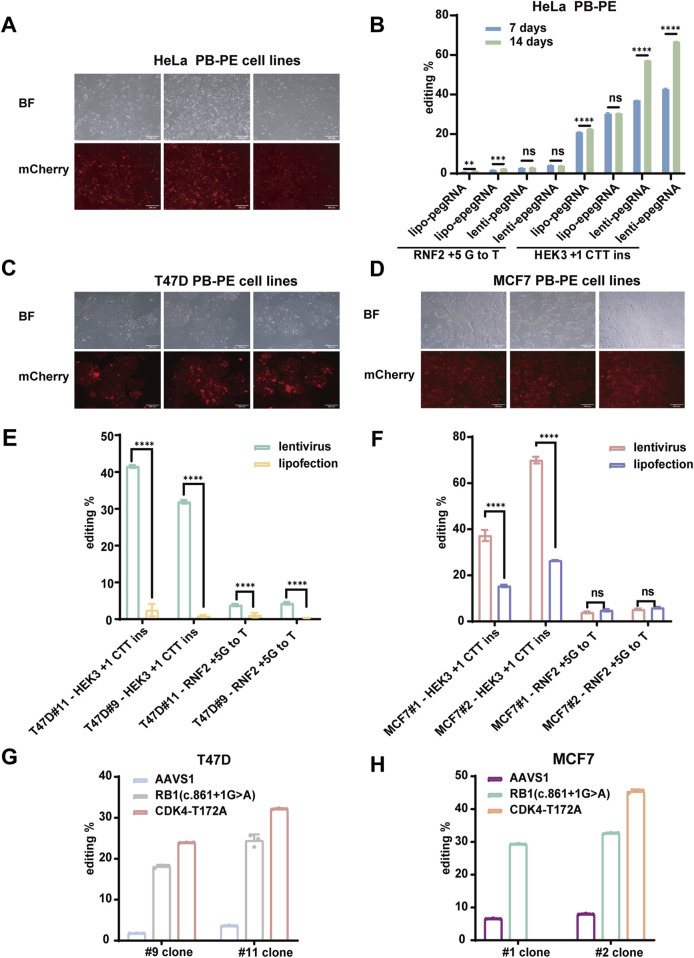
Efficient piggyBac Prime editing across multiple loci and cell lines. **(A)** mCherry fluorescence expression in HeLa PB-PE cell lines; scale bar, 200 µm. **(B)** PiggyBac prime editing of HEK3 and RNF2 loci in HeLa cells. **(C)** mCherry fluorescence expression in T47D PB-PE cell lines; scale bar, 200 µm. **(D)** mCherry fluorescence expression in MCF7 PB-PE cell lines; scale bar, 200 µm. **(E,F)** PiggyBac prime editing of HEK3 and RNF2 loci in T47D and MCF7 PB-PE clones. EpegRNAs were delivered via lipofectamine and lentiviral methods, respectively. The editing of T47D cells is shown in **(E)**, and the editing of MCF7 cells is shown in **(F)**. **(G)** Editing efficiency of the AAVS1, RB1, and CDK4 loci in two T47D PB-PE clones **(H)** Editing efficiency of the AAVS1, RB1, and CDK4 loci in two MCF7 PB-PE clones. Data are represented as mean ± SD (n = 3). P values were obtained using the 2-way ANOVA with Bonferroni multiple comparisons test. ** p < 0.01; *** p < 0.001; **** p < 0.0001; ns, p > 0.05.

We established a piggyBac-based prime editing (PB-PE) system in 293T cells, which significantly enhanced editing efficiency at the target sites. To evaluate the versatility of this system, we further validated PB-PE in various human cancer cell lines. First, we generated a stable PB-PE HeLa cell line and assessed its editing performance in pooled cells (Figure 3A). At the HEK3 locus, editing efficiency increased from 30.50% with transient transfection to 66.71% with lentiviral delivery of epegRNAs. However, editing efficiency at the RNF2 locus remained below 10% (Figure 3B), suggesting that prime editing efficiency may be influenced by factors such as genetic background, target site accessibility (Park et al., 2021; Liu N. et al., 2022), and mismatch repair (MMR) pathway activity (Chen et al., 2021; Ferreira da Silva et al., 2022).

Next, we validated the PB-PE system in the breast cancer cell lines T47D and MCF7. We generated two PB-PE single clones for each cell line (Figures 3C,D) and assessed editing efficiency at both the HEK3 and RNF2 loci. The average editing efficiency at the HEK3 locus was 41.51% for two T47D PB-PE single clones (Figure 3E) and 69.98% for two MCF7 PB-PE single clones (Figure 3F). Notably, MCF7 clone #2 consistently outperformed clone #1, while T47D clone #11 exhibited higher efficiency than clone #9, suggesting that variations in integration sites and prime editor expression levels among individual clones can influence editing efficiency. Additionally, unlike in 293T cells, editing efficiency at the RNF2 locus remained relatively low in MCF7, T47D, and HeLa cells, likely due to differences in the genetic backgrounds of these cell lines. Consistent with our findings in 293T cells, lentiviral delivery of epegRNAs led to improved editing efficiency compared to lipofection, highlighting the advantage of stable epegRNA expression for prime editing.

To further evaluate the performance of PB-PE, we assessed its editing efficiency at additional loci. Specifically, we introduced various single-base mutations to achieve amino acid substitutions using the PB-PE system. In T47D cells, the RB1 c.861 + 1G>A mutation was detected in 18.13% and 24.47% of sequenced reads for clones #9 and #11, respectively, while the CDK4 T172A (ACA>GCA) mutation accounted for 23.95% and 32.19% in clones #9 and #11, respectively (Figure 3G). Similarly, in MCF7 cells, the RB1 c.861 + 1G>A mutation was present in 29.32% and 32.66% of sequenced reads for clones #1 and #2, respectively, whereas the CDK4 T172A mutation reached 45.42% in MCF7 clone #2 (Figure 3H). Despite the substantial editing observed at the RB1 and CDK4 loci, base substitution at the AAVS1 locus exhibited lower efficiency (Figures 3G,H), likely due to sequence variations at the editing site and differences in pegRNA efficiency. Overall, our results demonstrate that PB-PE is effective across multiple cell lines and target loci, further highlighting its potential to enhance prime editing efficiency.

### 3.4 Robust piggyBac prime editing in human pluripotent stem cells

After demonstrating the excellent performance of PB-PE system in 293T cells and various human cancer cell lines, we next evaluated its efficacy in more challenging cell types. Human pluripotent stem cells (hPSCs) are particularly difficult to edit due to their low transfection efficiency, which has limited the widespread application of prime editing in these cells. Therefore, we aim to address this challenge and expand the potential of PB-PE for gene editing in hPSCs.

**FIGURE 4:**
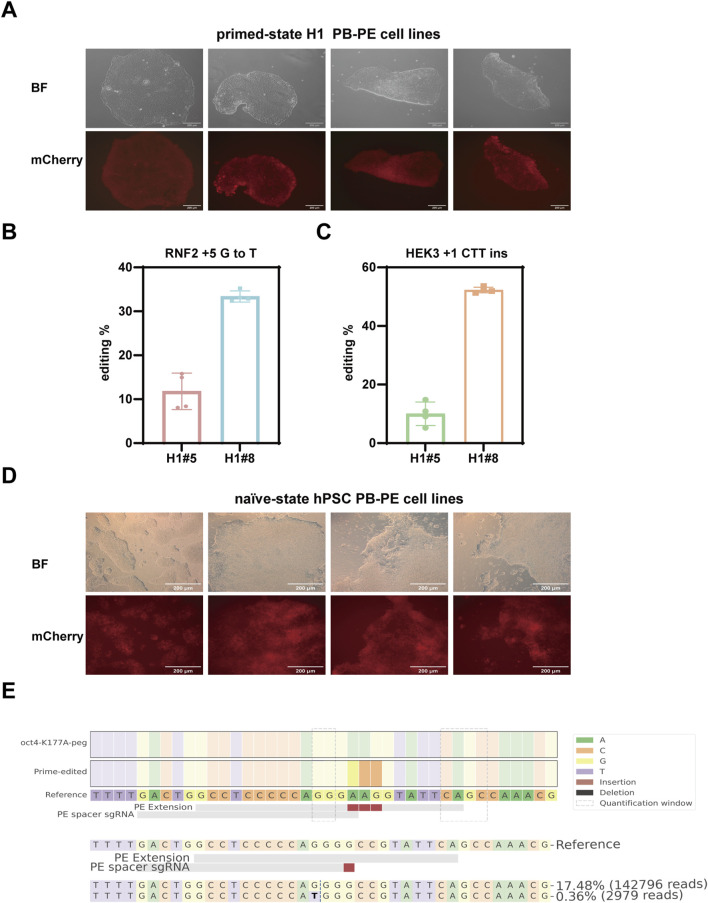
Prime editing using the PB-PE system in Human Pluripotent Stem Cells. **(A)** mCherry fluorescence expression in the primed-state H1 PB-PE cell lines; scale bar, 200 µm. **(B)** Editing efficiency of the RNF2 locus in two H1 clones. **(C)** Editing efficiency of the HEK3 locus in two H1 clones. Data are represented as mean ± SD (n = 4). **(D)** mCherry fluorescence expression in the naïve-state hPSC PB-PE cell lines; scale bar, 200 µm. **(E)** Editing efficiency of the OCT4 locus in naïve-state hPSC PB-PE cell lines.

We first established stable integration of the PB-PE system in the H1 human embryonic stem cell line (Figure 4A) and assessed its editing efficiency in two independent single clones. EpegRNAs were delivered via lentivirus, and editing efficiency was evaluated 7 days post-transduction. In clone #8, editing rates reached 52.27% at the HEK3 locus and 33.38% at the RNF2 locus, while clone #5 exhibited lower but still considerable editing efficiency at both loci (Figures 4B,C). These results highlight the strong potential of the PB-PE system in hPSCs and underscore the importance of selecting individual clones to achieve optimal performance.

Conventional hPSCs are considered to resemble the epiblast of the post-implantation human embryo *in vitro*. However, recent advancements have uncovered an alternative pluripotent state, known as the naïve state, which more accurately reflects the true starting point of human development (Nichols and Smith, 2009; Weinberger et al., 2016; Furlan et al., 2023; Zhou et al., 2023). Naïve hPSCs can be derived directly from preimplantation human embryos or reset from primed-state pluripotent stem cells *in vitro*. While our initial experiments were conducted in primed-state H1 cells, we further explored the performance of PB-PE in naïve-state hPSCs. We established stable PB-PE cell lines in the naïve state (Figure 4D) and targeted the key transcription factor OCT4, which is critical for maintaining pluripotency. NGS results revealed the successful introduction of a lysine-to-alanine substitution at position 177 in 17.48% of sequenced reads, with minimal unintended by-product edits (Figure 4E).

Overall, our results highlight the excellent performance of PB-PE in human pluripotent stem cells at both primed and naïve states. The system significantly enhances editing efficiency and demonstrates robust editing capability even in cells that are traditionally difficult to transfect. These findings further support PB-PE as a powerful tool for gene editing in stem cell research and regenerative medicine.

## 4 Discussion

By systematically optimizing our prime editing strategy, we achieved up to 80% editing efficiency across multiple loci and cell lines, including up to 50% efficiency in challenging cell types such as human pluripotent stem cells (hPSCs). Beyond improving the prime editing machinery itself, we focused on enhancing exogenous gene delivery to ensure strong, ubiquitous, and sustained expression of prime editing components. Key optimizations included stable genomic integration of prime editors using the piggyBac transposon system, selection of single-cell clones, utilization of an enhanced promoter, and lentiviral delivery of pegRNAs. Our approach significantly improved editing efficiency, increasing it from 23.37% to 81.78% using the same prime editor and epegRNAs. These findings highlight the critical role of optimizing prime editing component expression and demonstrate the potential of our strategy for broad applications in both basic research and therapeutic genome engineering.

Due to the large size of prime editing enzymes and the additional components required for optimal editing, such as hMLH1dn, conventional delivery methods like lentivirus and AAVs, which are widely used for CRISPR-Cas9, are unsuitable for prime editing (Liu P. et al., 2021; Böck et al., 2022; Gao et al., 2022; Liu B. et al., 2022; Zheng et al., 2022; Zhi et al., 2022). Instead, the piggyBac transposon system offers a superior solution, providing a large cargo capacity, stable genomic integration, and broad applicability across diverse cell types (Ding et al., 2005; Wilson et al., 2007; Chen et al., 2015; Yusa et al., 2015; Eggenschwiler et al., 2021; Wolff et al., 2021). However, since piggyBac-mediated integration occurs randomly with certain biases, selecting single-cell clones is essential to ensure precise integration and consistent expression of the prime editors.

Despite these advantages, generating stably integrated cell lines is a lengthy process, making it challenging to maintain sustained prime editor expression over time. We observed that the commonly used CMV promoter was suboptimal, as it is prone to silencing, particularly in stem cells, primary cells, and *in vivo* applications. In contrast, the CAG promoter demonstrated greater resistance to silencing, supporting long-term and stable gene expression. Moreover, the use of the CAG promoter is well-suited to the piggyBac system’s large cargo capacity, as it is significantly larger than the CMV promoter (1.6 kb vs 0.6 kb), making it a more robust choice for prime editing applications.

We also observed that sustained pegRNA expression significantly enhances editing efficiency. Consistent with previous reports, editing efficiency progressively increased from day 7 to day 14, suggesting a cumulative effect of prime editing over time, meaning that the longer the editing process continues, the higher the observed editing rates. While transient pegRNA transduction initially produces higher expression levels, lentiviral delivery ensures stable, long-term expression, ultimately leading to superior editing efficiency by day 14.

Although the continuous refinement of prime editing methodologies is beyond the scope of this study, such efforts remain essential for advancing the application of genome editing. One major challenge is the instability of the 3′end of pegRNA, which is prone to degradation (Liu Y. et al., 2021; Nelson et al., 2021; Feng et al., 2022; Li et al., 2022a; Liu B. et al., 2022; Zhang et al., 2022). Additionally, complementarity between the spacer and PBS sequences can lead to undesirable secondary structures, including intermolecular double-stranded formations and self-cyclization of the pegRNA, both of which can hinder editing efficiency (Liu B. et al., 2022; Ponnienselvan et al., 2023). Therefore, modifications to pegRNA are necessary to enhance its stability and functionality. Furthermore, editing efficiency is influenced by factors such as genetic background variations and target site preferences. Parameters including PBS and RTT length, as well as annealing temperature, can significantly impact editing outcomes (Lin et al., 2021; Nelson et al., 2021). Therefore, pegRNA design must be tailored to specific targets. The development of computational tools like PRIDICT 2.0 (Mathis et al., 2024) and DeepPrime (Yu et al., 2023) has improved the ability to predict editing efficiency, enabling more effective pegRNA design.

Editing efficiency is strongly influenced by the sequence and genomic locus-specific features. Despite the use of computational tools for pegRNA design, we still face significant challenges regarding the editing efficiency of certain gene loci. In our study, the RNF2 locus exhibited low editing efficiency in HeLa, MCF7, and T47D cells, whereas it showed a significantly different efficiency in 293T cells. Notably, even after optimization of the CAG promoter, the RNF2 locus remained inefficient specifically in these cell types. We hypothesize that this discrepancy may be attributed to differences in the genetic backgrounds of these cell lines, potentially involving factors such as variations in the MMR pathway and alterations in chromatin accessibility. Furthermore, the pegRNA scores at the RNF2 locus predicted by PRIDICT 2.0 were consistent with our speculation. The score was significantly higher in 293T cells (MMR-deficient cell line) compared to K562, HeLa, and other MMR-proficient cell lines. Thus, even high-scoring pegRNAs may exhibit variable editing efficiencies due to contextual factors such as cell line-specific effects.

In addition to pegRNA optimization, continuous improvements to the prime editor itself are crucial. Strategies such as optimizing nuclear localization signals (NLS) for better nuclear targeting (Chen et al., 2021; Liu P. et al., 2021), fusing chromatin-modifying peptides (CMP) to enhance chromatin accessibility (Park et al., 2021), and modifying reverse transcriptase (RT) structural domains have all contributed to enhanced editing efficiency (Song et al., 2021; Xu et al., 2021; Zong et al., 2022). Iterative improvements to the prime editor have led to the development of successive versions, progressing from PE1 to the latest PE7 (Chen et al., 2021; Eggenschwiler et al., 2021; Doman et al., 2023; Yan et al., 2024), each incorporating refinements that expand its applicability and precision.

The piggyBac transposon system exhibits stochastic integration, a feature that facilitates stable genomic integration and sustained expression of exogenous genes. However, the unpredictability of integration sites poses significant challenges to its safe application in gene therapy. For instance, random PB integration may disrupt endogenous gene regulation, leading to dysregulation of oncogenes or tumor suppressors, and potentially triggering oncogenesis. Therefore, we primarily utilize it as a tool for basic research to address the challenges of DNA delivery, thereby enabling the exploration of more efficient gene editing strategies and mechanistic studies, rather than for clinical applications in disease treatment. Nonetheless, several strategies can be employed to mitigate the risk of stochastic integration. Targeted integration approaches (Kettlun et al., 2011; Owens et al., 2012; Luo et al., 2017) can be employed to deliver exogenous genes into genomic safe harbors (GSHs), such as the AAVS1 and CCR5 locus. In addition, insertion sites can be monitored by whole genome sequencing. Similar to lentivirus-based therapies, piggyBac transposon-based therapies inherently raise safety concerns. Accordingly, the development of safety switches for systematic quality control of randomly integrated cells is crucial. The integration risks associated with the piggyBac transposon system can be mitigated through multiple strategies, thereby enhancing its safety in genome engineering.

Overall, our study provides a robust and efficient framework for prime editing, offering a promising strategy for applications in both fundamental research and therapeutic genome engineering. The advancements presented here lay the groundwork for further improvements in prime editing technologies, ultimately expanding their potential for precise and versatile genetic modifications.

## Data Availability

The original contributions presented in the study are included in the article, further inquiries can be directed to the corresponding author.
