# Supplementary material for: Systematic optimization of prime editing for enhanced efficiency and versatility in genome engineering across diverse cell types
Source: Front Cell Dev Biol. 2025 Apr 29;13:1589034. doi: 10.3389/fcell.2025.1589034 (PMC12069386; doi:10.3389/fcell.2025.1589034)
Supplement: Supplementary file 1 [file DataSheet1.pdf]

Supplementary Material

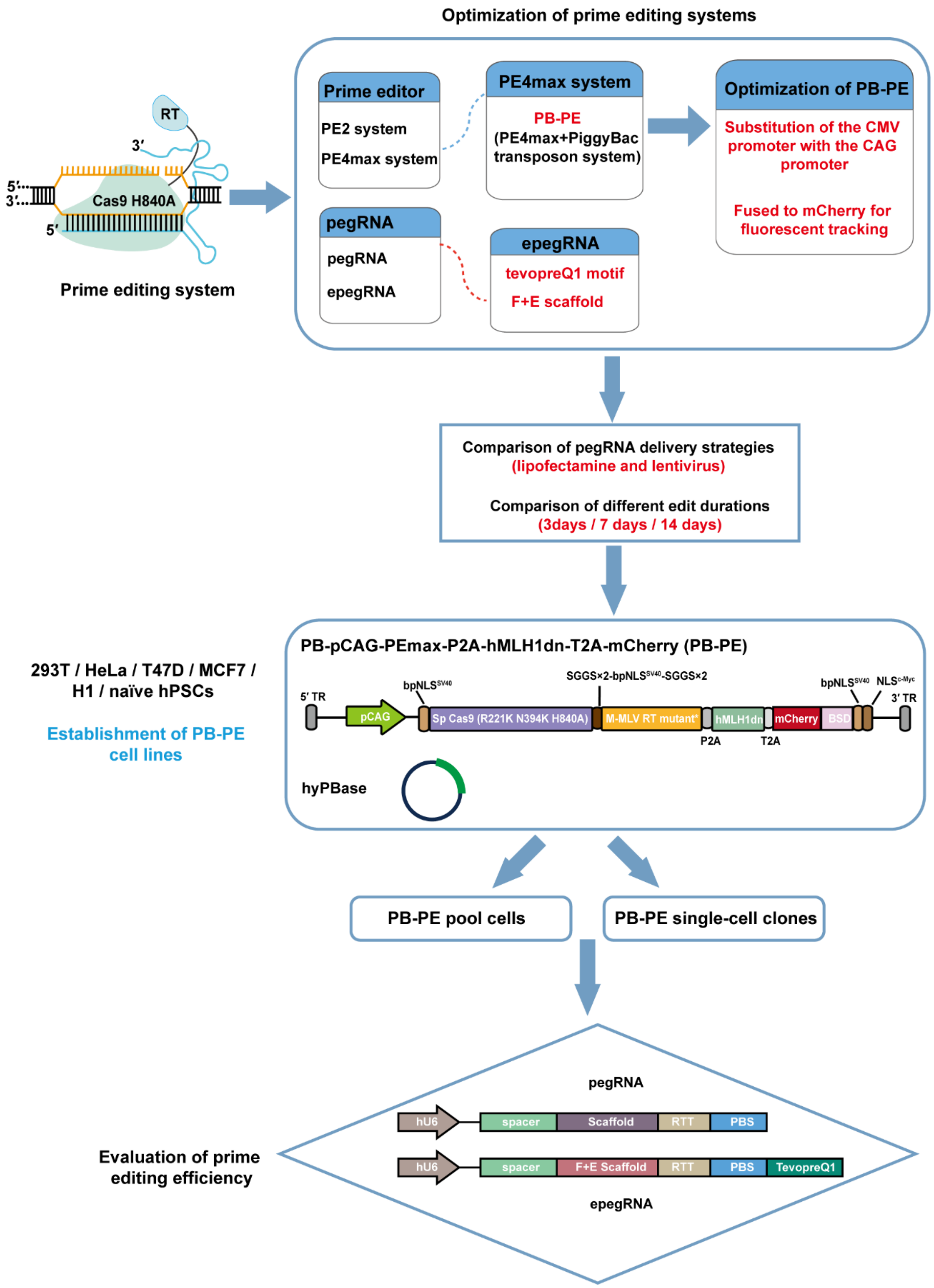

Supplementary Figure 1. Flowchart of the prime editing optimization strategy. The optimization of the prime editing system is categorized into prime editor and pegRNA element optimization. After systematic evaluation of both PE2 and PE4max architectures, the PE4max system was engineered for enhanced performance by associating with the piggyBac transposon, resulting in the development of the PB-PE system. The optimized PB-PE system was subsequently engineered through promoter replacement and mCherry reporter fusion. Engineered pegRNAs were created through incorporation of tevopreQ1 motif and scaffold replacement. We compared the effects of different pegRNA delivery methods and the impact of varying editing durations. Next, the PB-PE system was successfully established across multiple cell lines, with its editing efficiency was verified at multiple target loci.

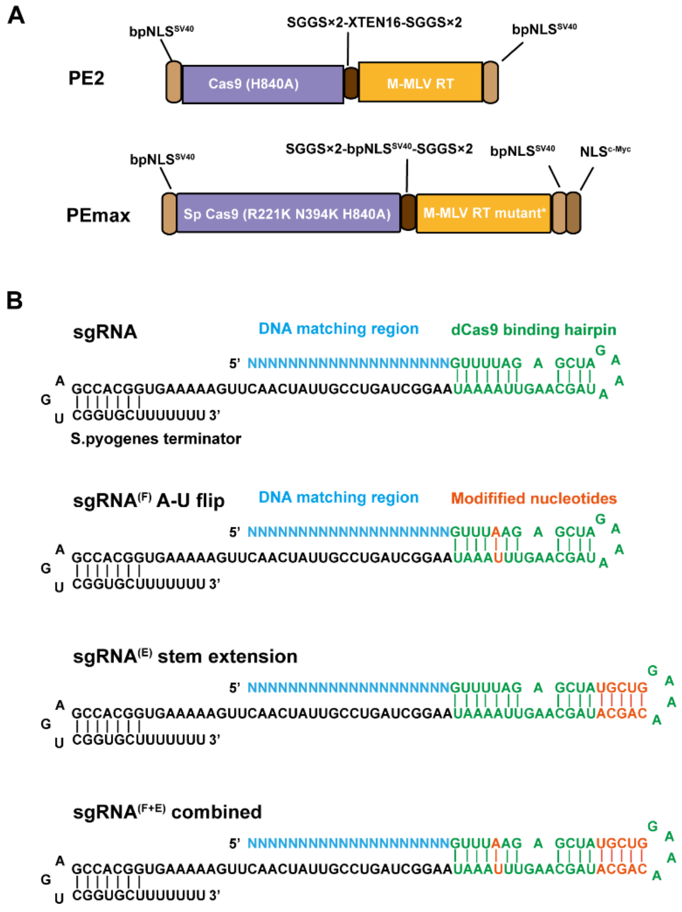

Supplementary Figure 2. A schematic representation of the prime editing system components. (**A**) Schematic representation of PE2 and PEmax. (**B**) Schematic representation of sgRNA scaffold and 'flip and extension' (F+E) sgRNA scaffold.

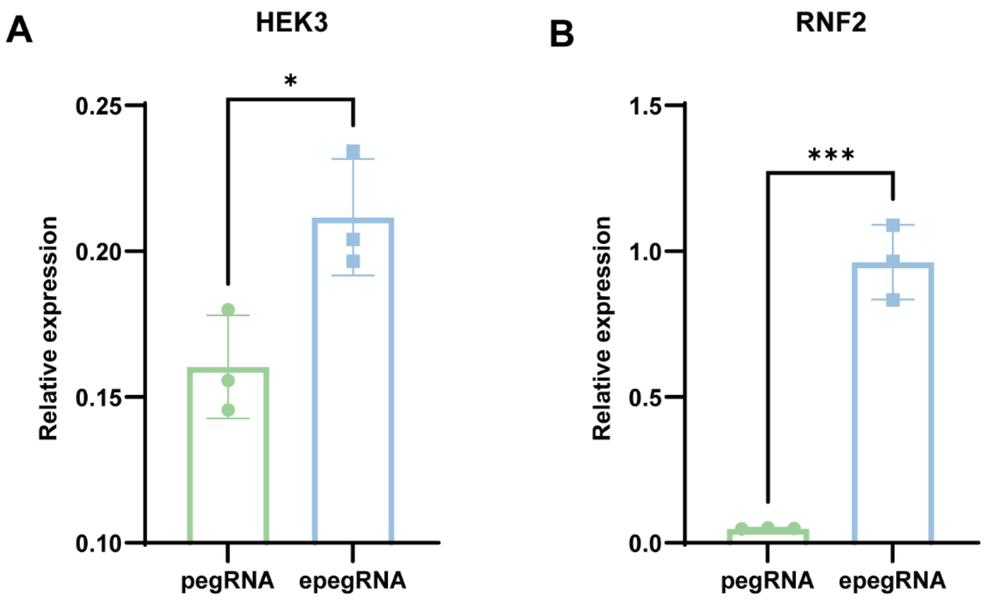

Supplementary Figure 3. Quantitative RT-qPCR analysis of pegRNA and epegRNA expression levels in the PE4max system. The expression levels of pegRNA and epegRNA were quantified separately at 7 days post-lentiviral transduction. (**A**) Quantitative RT-qPCR analysis of HEK3 locus. (**B**) Quantitative RT-qPCR analysis of RNF2 locus. Data are presented as mean ± SD from three independent experiments (n=3). Statistical significance was determined by Student's t-test (*p < 0.05, *p < 0.01). The relative expression levels (2−ΔCq) of pegRNA and epegRNA were compared between groups using an unpaired two-tailed Student's t-test. * p < 0.05; ** p < 0.01; *** p < 0.001; **** p < 0.0001; ns, p > 0.05.

Supplementary Table1. Plasmid construction primer

Description	Sequence (5'-3')	PCR fragment
pCAG-F	GGCCTCCACGGCCGACATTGATTATTGACTAGTTATTAATAGTAATCAATTACGGGGTCA	CAG promoter 1741 bp
CAG-R	GAATTCTTTGCCAAAATGAT	
T7-cas9-F	GTCTCATCATTTTGGCAAAGAATTCTAATACGACTCACTATAGGGA	T7-SV40 NLS-Cas9 141 bp
SV40NLS-Cas9-R	GTCCAGGCCGATGCTGTACTTCTTGTCGACTTTCCGCTTCTTCTTTGGT	
T2A-mcherry-F	CCTGCCCGACCTTTATAAGGTTTTCGGCAGTGGAGAGGGCAGAGG	T2A-mCherry 828bp
mcherry-pmeI-R	GTGTTTCAGTTAGCCTCCCCCGTTTAAACTTACTTGTACAGCTCGTCCA	
MLH1dn-sbfi-F	TCATCAACCTGACCTCCGTGCTGAGCCTGCAGGAGGAGATCAACGAGCAGGGC	hMLH1dn fragment 761 bp
MLH1dn-R	GAAAACCTTATAAAGGTCGG	
pCAG-AgeI-Kozak-hyPBase-F	CGACGGTACCGCGGGCCCGGGATCCACCGGTCGCCACCATGGGAA	hyPBase fragment 1857 bp
hyPBase-NotI-pCAG-R	GATAGGCAGCCTGCACCTGAGGAGTGCGGCCGCTTTAGAAACAGGACTGGCACATGT	
Align-LGP-NsiI-F	ACATTTTTAAATGTTTCATCTTCAAATGCAT	region1 fragment 202 bp
LGP-BsmBI- tevopreQ1-R	TTCTAGTTGGTTTAACGCGTAACTAGATAGAACCGCGAGAGACGTACAAAAAAGAGCAAGAAGC	
tevopreQ1-T6-LGP-align-F	CGCGGTTCTATCTAGTTACGCGTTAAACCAACTAGAATTTTTTAAGCTTGGCGTAACTAGATCTTGAG	region2 fragment 293 bp
Align-LGP-XmaI-R	GTTTAAAACTTTATCCATCTTTGCACCCGGG	
F+E-scaffold-F	GTTTAAGAGCTAAGCTGGAAACAGCATAGCAAGTTTAAATAAGGCTAGTCCGTTATCAAC	F+E-scaffold 86 bp
F+E-scaffold-R	AGCACCGACTCGGTGCCACTTTTTCAAGTTGATAACGGACTAGCCTTATTTAAACTTGCTA	
HEK3-CTTins-gSP_LV_F	TATATATCTTGTGGAAAGGACGAAACACCGGGCCCAGACTGAGCACGTGAGTTTAAGAGCTAAGCTGGAA	HEK3 epegRNA 193 bp
HEK3-CTTins _LV_R	TGGTTTAACGCGTAACTAGATAGAACCGCGCAGACTGAGCACGCTTTGATGGCAGAAGCACCGACTCGGTGCCACTTTTTCA	
RNF2-gSP_LV_F	TATATATCTTGTGGAAAGGACGAAACACCGGTCATCTTAGTCATTACCTGGTTTAAGAGCTAAGCTGGAA	RNF2 epegRNA 197bp
RNF2-Tevopre_LV_R	TGGTTTAACGCGTAACTAGATAGAACCGCGCATCTTAGTCATTACCTGATGTGTTCGTTAGCACCGACTCGGTGCCACTTTTTCA	
CDK4-gSP_LV_F	TATATATCTTGTGGAAAGGACGAAACACCGACCATCTTTCTACTGACCACGTTTAAGAGCTAAGCTGGAA	CDK4 epegRNA 199 bp
CDK4-Tevopre_LV_R	TGGTTTAACGCGTAACTAGATAGAACCGCGCTTTCTACTGACCACGGGTGCAAGTGCCATCTAGCACCGACTCGGTGCCACTTTTTCA	
AAVS1-gSP_LV_F	TATATATCTTGTGGAAAGGACGAAACACCGGAGGGGCTCAACATCGGAAGGTTTAAGAGCTAAGCTGGAA	AAVS1 epegRNA 194bp
AAVS1-Tevopre_LV_R	TGGTTTAACGCGTAACTAGATAGAACCGCGGCTCAACATCGGAAGAGGAGAAGTCGAAGCACCGACTCGGTGCCACTTTTTCA	
RB1-gSP_LV_F	TATATATCTTGTGGAAAGGACGAAACACCGACATGAATGTAATATAGATGGTTTAAGAGCTAAGCTGGAA	RB1 epegRNA 193 bp
RB1-Tevopre_LV_R	TGGTTTAACGCGTAACTAGATAGAACCGCGTGAATGTAATATAGATGAGATAATTTAGCACCGACTCGGTGCCACTTTTTCA	
OCT4-gSP_LV_F	TATATATCTTGTGGAAAGGACGAAACACCGGGACTGGCCTCCCCCAGGGAGTTTAAGAGCTAAGCTGGAA	OCT4 epegRNA 190 bp
OCT4-Tevopre_LV_R	TGGTTTAACGCGTAACTAGATAGAACCGCGGCCTCCCCCAGGGGCCGTATTCAAGCACCGACTCGGTGCCACTTTTTCA	

Supplementary Table2. pegRNA sequence

pegRNA	spacer sequence	3' extension	PBS length (nt)	RTT length (nt)
HEK3 + 1 CTT ins	GGCCCAGACTGAGCACGTGA	TCTGCCATCAAAGCGTGCTCAGTCTG	14	12
RNF2 + 5 G to T	GTCATCTTAGTCATTACCTG	AACGAACACATCAGGTAATGACTAAGATG	15	14
CDK4 T172A	ACCATCTTTCTACTGACCAC	AGATGGCACTTGCACCCGTGGTCAGTAGAAAG	12	20
AAVS1_55116850_1_G_1_A	GAGGGGCTCAACATCGGAAG	TCGACTTCTCCTCTTCCGATGTTGAGC	12	15
RB1(c.861+1 G>A)	ACATGAATGTAATATAGATG	AAATTATCTCATCTATATTACATTCA	14	12
OCT4 K177A	GGACTGGCCTCCCCCAGGGA	TGAATACGGCCCCTGGGGGAGGC	14	9

Construction of pB-pCMV-PEmax-P2A-hMLH1dn vector

Step 1: Digest pCMV-PEmax-P2A-hMLH1dn plasmid

pCMV-PEmax-P2A-hMLH1dn plasmid	1.5 μg
SpeI-HF(NEB)	1.5 μL
PmeI (NEB)	1.5 μL
10× rCutSmart Buffer	5.0 μL
ddH_2O	to 50.0 μL
Total reaction volume	50.0 μL

Incubate at 37 °C for 3 hours

Isolate 9,390 bp fragment from cut plasmid

Step 2: Digest pB-CAGGS-dCas9-KRAB-MeCP2 plasmid

pB-CAGGS-dCas9-KRAB-MeCP2 plasmid	1.5 μg
SpeI-HF(NEB)	1.5 μL
PmeI (NEB)	1.5 μL
10× rCutSmart Buffer	5.0 μL
ddH_2O	to 50.0 μL
Total reaction volume	50.0 μL

Incubate at 37 °C for 3 hours

Isolate 5,772 bp fragment from cut plasmid

Step 3: Ligate the linearized plasmid

pCMV-PEmax-P2A-hMLH1dn fragment (9,390 bp)	1.0 μL
pB-CAGGS-dCas9-KRAB-MeCP2 fragment (5,772 bp)	1.0 μL
2×T4 DNA Ligase Buffer (NEB)	2.0 μL
T4 DNA Ligase (NEB)	1.0 μL
ddH_2O	to 20.0 μL
Total reaction volume	20.0 μL

Incubate at 16 °C overnight

Transform the ligation product into competent cells. Cells transformed with the assembled plasmid were selected using ampicillin resistance.

Construction of pB-pCAG-PEmax-P2A-hMLH1dn vector

Step 1: Digest PB-pCMV-PEmax-P2A-hMLH1dn plasmid

PB-pCMV-PEmax-P2A-hMLH1dn plasmid	1.0 µg
SpeI-HF (NEB)	1.5 µL
SalI-HF (NEB)	1.5 µL
10× rCutSmart Buffer	5.0 µL
ddH_2O	to 50.0 µL
Total reaction volume	50.0 µL

Incubate at 37 °C for 3 hours
Isolate 14,465 bp fragment from cut plasmid

Step 2: PCR amplification of the CAG promoter and T7-SV40 NLS-Cas9 fragments

pCAG-hyPBase plasmid	20.0 ng
pCAG-F (10 µM)	1.0 µL
CAG-R (10 µM)	1.0 µL
2×KapaHIFI	25.0 µL
ddH_2O	to 50.0 µL
Total reaction volume	50.0 µL

The PCR program was as follows: 95 °C for 3 minutes, followed by 30 cycles of (98 °C for 20 seconds, 55 °C for 15 seconds, 72 °C for 1 minute), with a final extension at 72 °C for 5 minutes, and hold at 4 °C. The amplified CAG promoter fragment was 1,741 bp.

pCMV-PEmax-P2A-hMLH1dn plasmid	20.0 ng
T7-cas9-F (10 µM)	1.0 µL
SV40NLS-Cas9-R (10 µM)	1.0 µL
2×KapaHIFI	25.0 µL
ddH_2O	to 50.0 µL
Total reaction volume	50.0 µL

The PCR program was as follows: 95°C for 3 minutes, followed by 30 cycles of (98 °C for 20 seconds, 65 °C for 15 seconds, 72 °C for 15 seconds), with a final extension at 72 °C for 5 minutes, and hold at 4 °C. The amplified T7-SV40 NLS-Cas9 fragment was 141 bp.

Step 3: Ligate the linearized vector and PCR fragments

Digested PB-pCMV-PEmax-P2A-hMLH1dn plasmid (14465 bp)	1.0 µL
CAG promoter fragment	1.0 µL
T7-SV40 NLS-Cas9 fragment	1.0 µL
2×MultiF Seamless Assembly Mix (Abclonal)	5.0 µL
ddH_2O	to 10.0 µL
Total reaction volume	10.0 µL

Incubate at 50 °C for 30 minutes
Transform the ligation product into competent cells. Cells transformed with the assembled plasmid were selected

using ampicillin resistance.

Construction of pB-pCAG-PEmax-P2A-hMLH1dn-T2A-mCherry vector

Step 1: Digest pCAG-PEmax-P2A-hMLH1dn plasmid

PB-pCAG-PEmax-P2A-hMLH1dn plasmid	1.0 μg
PmeI (NEB)	1.0 μL
SbfI-HF (NEB)	1.0 μL
10x rCutSmart Buffer	5.0 μL
ddH_2O	to 50.0 μL
Total reaction volume	50.0 μL

Incubate at 37 °C for 3 hours
Isolate 15,502 bp fragment from cut plasmid

Step 2: PCR amplification of the T2A-mCherry and hMLH1dn fragments

Lenti-dCas9-Zim3-mCherry plasmid	20.0 ng
T2A-mCherry-F (10 μM)	1.0 μL
mCherry-PmeI-R (10 μM)	1.0 μL
2×KapaHIFI	25.0 μL
ddH_2O	to 50.0 μL
Total reaction volume	50.0 μL

The PCR program was as follows: 95°C for 3 minutes, followed by 30 cycles of (98 °C for 20 seconds, 65 °C for 15 seconds, 72 °C for 30 seconds), with a final extension at 72 °C for 5 minutes, and hold at 4 °C. The amplified T2A-mCherry fragment was 828 bp.

pB-pCAG-PEmax-P2A-hMLH1dn plasmid	20.0 ng
MLH1dn-SbfI-F (10 μM)	1.0 μL
MLH1dn-R (10 μM)	1.0 μL
2×KapaHIFI	25.0 μL
ddH_2O	to 50.0 μL
Total reaction volume	50.0 μL

The PCR program was as follows: 95°C for 3 minutes, followed by 30 cycles of (98 °C for 20 seconds, 50 °C for 15 seconds, 72 °C for 30 seconds), with a final extension at 72 °C for 5 minutes, and hold at 4 °C. The amplified hMLH1dn fragment was 761 bp.

Step 3: Ligate the linearized vector and PCR fragments

Digested PB-pCAG-PEmax-P2A-hMLH1dn plasmid (9730 bp)	1.0 μL
hMLH1dn fragment	1.0 μL
T2A-mcherry fragment	1.0 μL
2×MultiF Seamless Assembly Mix (Abclonal)	5.0 μL
ddH_2O	to 10.0 μL
Total reaction volume	10.0 μL

Incubate at 50 °C for 30 minutes
Transform the ligation product into competent cells. Cells transformed with the assembled plasmid were selected

using ampicillin resistance.

Construction of pCAG-hyPBase vector

Step 1: Digest CAG-CBE4max-SpG-P2A-EGFP plasmid

CAG-CBE4max-SpG-P2A-EGFP plasmid	1.0 µg
AgeI-HF (NEB)	1.0 µL
NotI-HF (NEB)	1.0 µL
10x rCutSmart Buffer	5.0 µL
ddH_2O	to 50.0 µL
Total reaction volume	50.0 µL

Incubate at 37 °C for 3 hours
Isolate 4,826 bp fragment from cut plasmid

Step 2: PCR amplification of the hyPBase fragment

AgeI-KOZAK-hyPBase-NotI synthesis template	20.0 ng
pCAG-AgeI-Kozak-hyPBase-F (10 µM)	1.0 µL
hyPBase-NotI-pCAG-R (10 µM)	1.0 µL
2×KapaHIFI	25.0 µL
ddH_2O	to 50.0 µL
Total reaction volume	50.0 µL

The PCR program was as follows: 95°C for 3 minutes, followed by 30 cycles of (98 °C for 20 seconds, 65 °C for 15 seconds, 72 °C for 1 minute), with a final extension at 72 °C for 5 minutes, and hold at 4 °C. The amplified hyPBase fragment was 1,857 bp.

Step 3: Ligate the linearized vector and PCR fragments

Digested CAG-CBE4max-SpG-P2A-EGFP plasmid (4826 bp)	1.0 µL
hyPBase fragment	1.0 µL
2×MultiF Seamless Assembly Mix (Abclonal)	5.0 µL
ddH_2O	to 10.0 µL
Total reaction volume	10.0 µL

Incubate at 50 °C for 30 minutes
Transform the ligation product into competent cells. Cells transformed with the assembled plasmid were selected using ampicillin resistance.

Construction of pegRNAs

Step 1: Construction of Lenti-TevopreQ1-Puro backbone vector
Digest Lenti-guide-puro vector plasmid

Lenti-guide-puro vector plasmid	1.5 μg
NsiI-HF (NEB)	1.0 μL
XmaI (NEB)	1.0 μL
10x rCutSmart Buffer	5.0 μL
ddH_2O	to 50.0 μL
Total reaction volume	50.0 μL

Incubate at 37 °C for 3 hours
Isolate 9,746 bp fragment from cut plasmid

PCR amplification of the region1 and region2 fragments

Lenti-guide-puro vector plasmid	20.0 ng
Align-LGP-NsiI-F (10 μM)	1.0 μL
LGP-BsmBI- tevopreQ1-R (10 μM)	1.0 μL
2×KapaHIFI	25.0 μL
ddH_2O	to 50.0 μL
Total reaction volume	50.0 μL

The PCR program was as follows: 95°C for 3 minutes, followed by 30 cycles of (98 °C for 20 seconds, 55 °C for 15 seconds, 72 °C for 15 seconds), with a final extension at 72 °C for 5 minutes, and hold at 4 °C. The amplified region1 fragment was 202 bp.

Lenti-guide-puro vector plasmid	20.0 ng
tevopreQ1-T6-LGP-align-F (10 μM)	1.0 μL
Align-LGP-XmaI-R (10 μM)	1.0 μL
2×KapaHIFI	25.0 μL
ddH_2O	to 50.0 μL
Total reaction volume	50.0 μL

The PCR program was as follows: 95°C for 3 minutes, followed by 30 cycles of (98 °C for 20 seconds, 60 °C for 15 seconds, 72 °C for 15 seconds), with a final extension at 72 °C for 5 minutes, and hold at 4 °C. The amplified region2 fragment was 293 bp.

Ligate the linearized vector and PCR fragments

Digested Lenti-guide-puro vector plasmid (9746 bp)	1.0 μL
region1	1.0 μL
region2	1.0 μL
2×MultiF Seamless Assembly Mix (Abclonal)	5.0 μL
ddH_2O	to 10.0 μL
Total reaction volume	10.0 μL

Incubate at 50 °C for 30 minutes
Transform the ligation product into competent cells. Cells transformed with the assembled plasmid were selected

using ampicillin resistance.

Step 2: Digest Lenti-TevopreQ1-Puro plasmid

Lenti-TevopreQ1-Puro plasmid	1.5 µg
BsmBI-v2 (NEB)	2.0 µL
r3.1 buffer (NEB)	5.0 µL
ddH$_2$O	to 50.0 µL
Total reaction volume	50.0 µL

Incubate at 55 °C for 3 hours
Isolate 8,259 bp fragment from cut plasmid

Step 3: Generate the F+E scaffold fragment

F+E-scaffold-F (10 µM)	1.0 µL
F+E-scaffold-R (10 µM)	1.0 µL
2×KapaHIFI	25.0 µL
ddH$_2$O	to 50.0 µL
Total reaction volume	50.0 µL

The PCR program was as follows: 95°C for 3 minutes, followed by 30 cycles of (98 °C for 20 seconds, 65 °C for 15 seconds, 72 °C for 15 seconds), with a final extension at 72 °C for 5 minutes, and hold at 4 °C. The amplified F+E scaffold fragment was 86 bp.

Step 4: Generate the epeg guide cloning insert using the F+E scaffold fragment as the PCR template

F+E scaffold fragment	20.0 ng
gSP-LV-F (10 µM)	1.0 µL
Tevopre-LV-R (10 µM)	1.0 µL
2×KapaHIFI	25.0 µL
ddH$_2$O	to 50.0 µL
Total reaction volume	50.0 µL

The PCR program was as follows: 95°C for 3 minutes, followed by 30 cycles of (98 °C for 20 seconds, 65 °C for 15 seconds, 72 °C for 15 seconds), with a final extension at 72 °C for 5 minutes, and hold at 4 °C. The amplified HEK3 fragment was 193 bp (RNF2 fragment was 197 bp, AAVS1 fragment was 194 bp, CDK4 fragment was 199 bp, RB1 fragment was 193 bp, OCT4 fragment was 190 bp).

Step 5: pegRNA assembly

Digested Lenti-TevopreQ1-Puro plasmid (8,259 bp)	1.0 µL
epeg guide cloning insert	1.0 µL
2×MultiF Seamless Assembly Mix (Abclonal)	5.0 µL
ddH$_2$O	to 10.0 µL
Total reaction volume	10.0 µL

Incubate at 50 °C for 30 minutes

Transform the ligation product into competent cells. Cells transformed with the assembled plasmid were selected using ampicillin resistance.

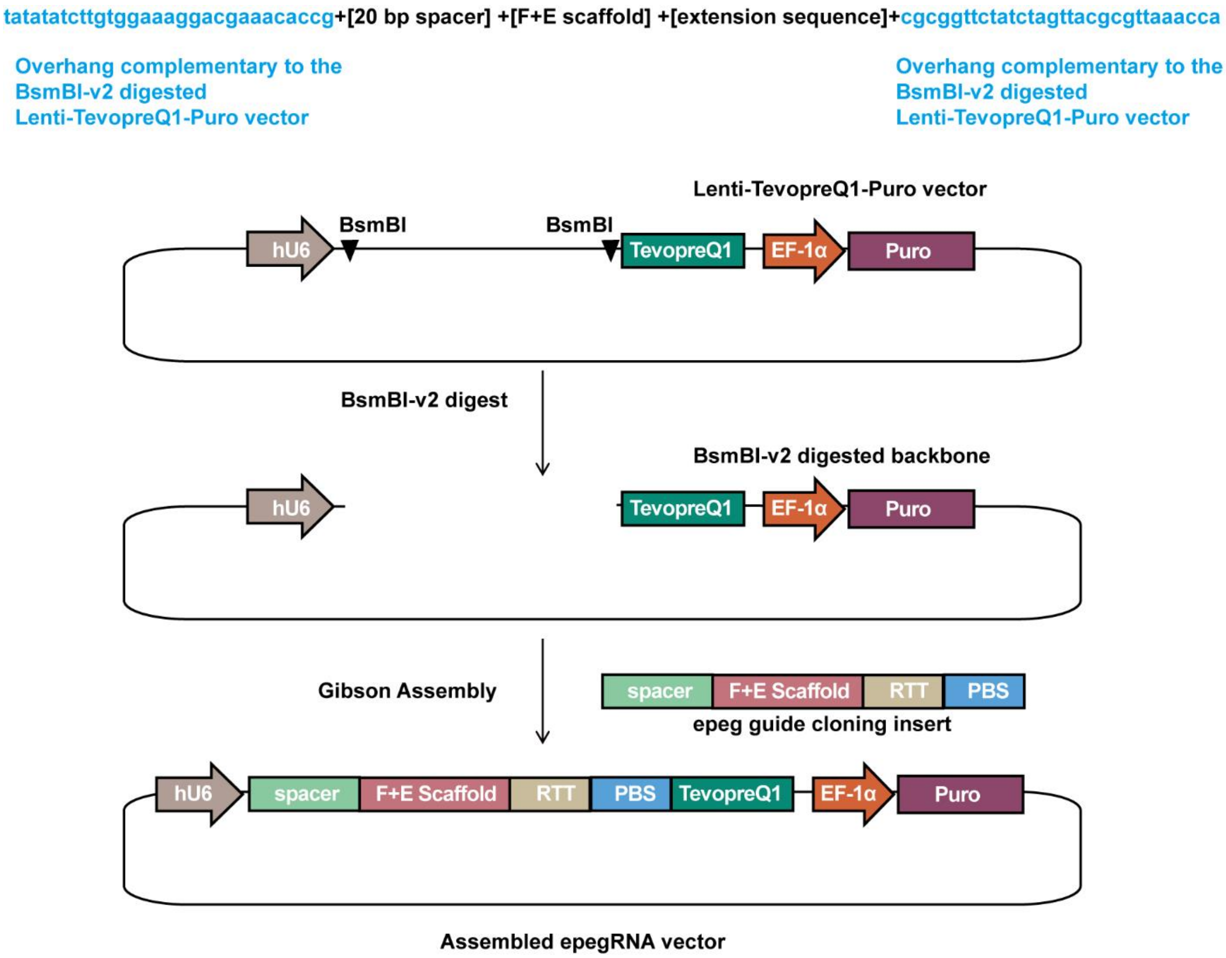

Supplementary Figure 4. Schematic representation of epegRNA construction. (1) BsmBI-v2 digested the Lenti-TevopreQ1-Puro vector. (2) PCR amplification of epeg guide cloning insert fragment with homology arms. (3) The BsmBI-digested Lenti-TevopreQ1-Puro vector was assembled with the PCR-amplified epegRNA fragment via homologous recombination.

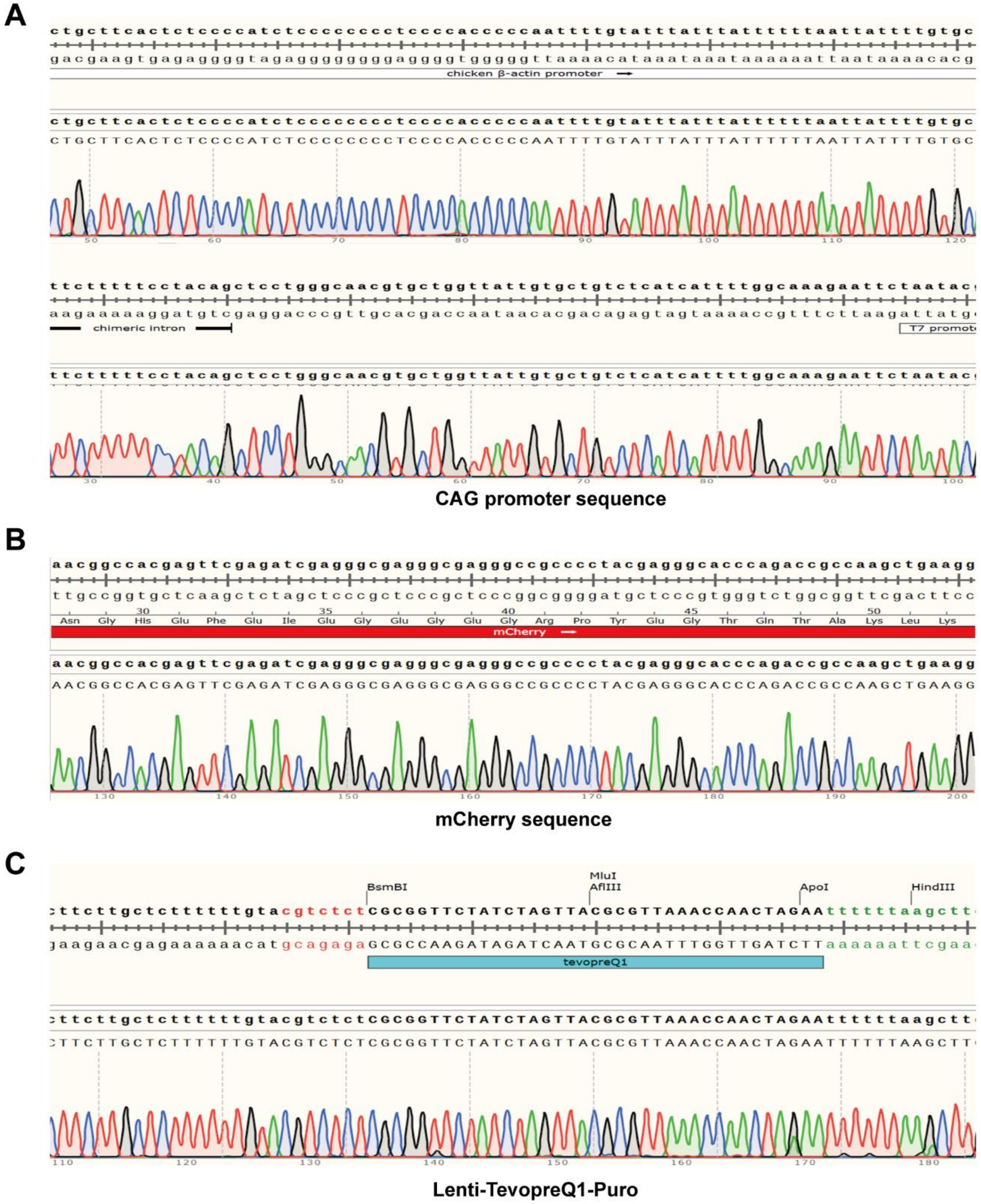

Supplementary Figure 5. Sequencing results of plasmid construction. (**A**) Results of Sanger sequencing for the pB-pCAG-PEmax-P2A-hMLH1dn vector. (**B**) Results of Sanger sequencing for the pB-pCAG-PEmax-P2A-hMLH1dn-T2A-mCherry vector. (**C**) Results of Sanger sequencing for the Lenti-TevopreQ1-Puro vector.

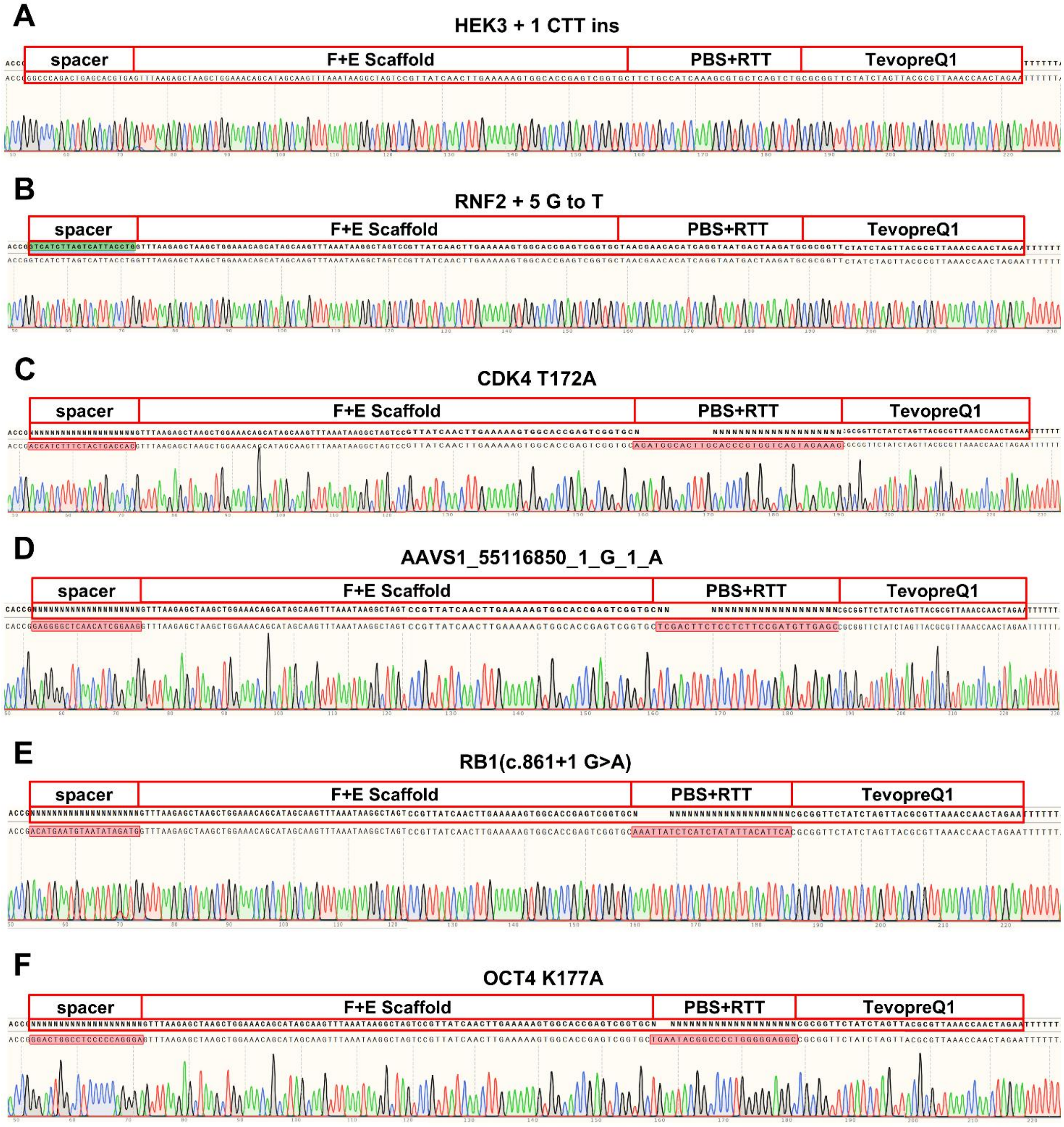

Supplementary Figure 6. Sanger sequencing results for epegRNA constructs. Figures A-F show epegRNA Sanger sequencing results for HEK3 (**A**), RNF2 (**B**), CDK4 (**C**), AAVS1 (**D**), RB1 (**E**), and OCT4 (**F)** loci.

AgeI-KOZAK-hyPBase-NotI synthesis template

ACCGGTCGCCACCATGGGAAGCTCACTCGATGACGAACATATCTTGTCCGCTTTGCTCCAAAGCGATGATGAACTGGTGGGAGAAGATT
CTGATAGTGAGGTGAGCGACCACGTGTCAGAAGATGACGTACAGTCTGACACAGAGGAGGCGTTCATAGATGAGGTCCATGAGGTCCA
ACCTACATCTAGCGGATCTGAGATCCTGGACGAACAGAACGTAATTGAACAACCTGGAAGCAGTTTGGCCAGCAACAGGATCCTTACTC
TTCCTCAAAGGACAATTAGGGGAAAGAACAAGCACTGCTGGTCCACATCTAAACCTACAAGGAGAAGTCGAGTGAGCGCACTCAACAT
AGTAAGATCTCAAAGGGGGCCTACTAGGATGTGTAGAAATATCTACGACCCGCTCCTGTGCTTCAAGCTCTTTTTCACTGACGAGATCAT
AAGCGAGATCGTCAAGTGGACAAACGCCGAGATCAGTCTCAAAAGAAGAGAGAGCATGACCTCCGCGACATTTAGGGACACGAATGA
GGACGAGATCTACGCTTTCTTTGGCATCCTGGTCATGACTGCCGTCAGGAAGGACAACCATATGTCTACAGACGACTTGTTCGATCGGTC
CCTTTCTATGGTCTACGTCAGCGTTATGAGCCGGGATCGATTCGATTTCCTGATCCGGTGCCTGAGGATGGATGACAAGTCCATCAGACC
GACACTCCGAGAGAACGATGTATTCACACCCGTGAGGAAGATCTGGGACCTGTTCATCCACCAGTGCATCCAGAATTATACACCGGGGG
CACACCTGACTATCGACGAACAATTGCTCGGATTTAGAGGACGCTGTCCTTTTCGCGTCTACATCCCTAACAAACCGAGCAAGTACGGA
ATCAAGATCCTCATGATGTGCGACTCCGGTACGAAGTACATGATCAACGGGATGCCTTATTTGGGTAGAGGAACACAAACAAACGGTGT
CCCTCTCGGTGAGTACTACGTGAAGGAACTCTCCAAGCCAGTCCACGGTTCATGCAGGAACATCACATGCGATAACTGGTTCACTTCCA
TACCCCTTGCCAAAAATCTCCTCCAAGAACCCTACAAACTGACCATCGTTGGAACTGTACGGTCCAATAAGAGGGAAATCCCGGAGGTC
TTGAAGAACTCAAGAAGCAGACCAGTTGGCACCTCAATGTTTTGCTTTGATGGCCCACTCACACTGGTCAGTTACAAGCCAAAACCGGC
GAAAATGGTGTACCTGCTTAGTAGCTGCGATGAAGACGCATCTATAAATGAGAGTACCGGAAAGCCACAGATGGTCATGTACTACAAC
CAGACAAAAGGGGGGGTGGACACTTTGGATCAGATGTGCAGCGTTATGACCTGCAGTAGGAAAACAAATCGCTGGCCAATGGCACTCC
TCTACGGTATGATCAACATCGCCTGCATCAACTCCTTCATTATCTACTCCCACAACGTCAGCTCAAAGGGTGAGAAGGTCCAGTCCCGG
AAGAAATTCATGAGGAACCTCTATATGGGGCTGACCTCATCTTTTATGCGCAAACGCCTTGAAGCACCTACACTCAAGAGGTACCTCAG
GGACAACATCTCCAACATTCTCCCCAAGGAAGTTCCAGGTACTTCTGACGACTCAACCGAAGAGCCAGTGATGAAGAAACGCACATACT
GCACCTATTGCCCGTCCAAGATTAGACGCAAGGCATCCGCTAGCTGCAAGAAATGCAAGAAGGTCATCTGTCGGGAGCATAACATCGA
CATGTGCCAGTCCTGTTTCTAGAGCGGCCGC

pU6-epegRNA vector

U6 promoter-Spacer-F+E scaffold-3’ extension (RTT+PBS)-TevopreQ1-Plasmid backbone

GAGGGCCTATTTCCCATGATTCCTTCATATTTGCATATACGATACAAGGCTGTTAGAGAGATAATTAGAATTAATTTGACTGTAAACACA
AAGATATTAGTACAAAATACGTGACGTAGAAAGTAATAATTTCTTGGGTAGTTTGCAGTTTTAAAATTATGTTTTAAAATGGACTATCAT
ATGCTTACCGTAACTTGAAAGTATTTCGATTTCTTGGCTTTATATATCTTGTGGAAAGGACGAAACACCGNNNNNNNNNNNNNNNNNNN
NGTTTAAGAGCTAAGCTGGAAACAGCATAGCAAGTTTAAATAAGGCTAGTCCGTTATCAACTTGAAAAAGTGGCACCGAGTCGGTGCN
NNNNNNNNNNNNNNNNNNNNCGCGGTTCTATCTAGTTACGCGTTAAACCAACTAGAATTTTTTAAGCTTGGCGTAACTAGATCTTGAGA
CAAATGGCAGTATTCATCCACAATTTTAAAAGAAAAGGGGGGATTGGGGGGTACAGTGCAGGGGAAAGAATAGTAGACATAATAGCA
ACAGACATACAAACTAAAGAATTACAAAAACAAATTACAAAAATTCAAAATTTTCGGGTTTATTACAGGGACAGCAGAGATCCACTTT
GGCGCCGGCTCGAGGGGGCCCGGGTGCAAAGATGGATAAAGTTTTAAACAGAGAGGAATCTTTGCAGCTAATGGACCTTCTAGGTCTT
GAAAGGAGTGGGAATTGGCTCCGGTGCCCGTCAGTGGGCAGAGCGCACATCGCCCACAGTCCCCGAGAAGTTGGGGGGAGGGGTCGGC
AATTGATCCGGTGCCTAGAGAAGGTGGCGCGGGGTAAACTGGGAAAGTGATGTCGTGTACTGGCTCCGCCTTTTTCCCGAGGGTGGGGG
AGAACCGTATATAAGTGCAGTAGTCGCCGTGAACGTTCTTTTTCGCAACGGGTTTGCCGCCAGAACACAGGTAAGTGCCGTGTGTGGTT
CCCGCGGGCCTGGCCTCTTTACGGGTTATGGCCCTTGCGTGCCTTGAATTACTTCCACCTGGCTGCAGTACGTGATTCTTGATCCCGAGC
TTCGGGTTGGAAGTGGGTGGGAGAGTTCGAGGCCTTGCGCTTAAGGAGCCCCTTCGCCTCGTGCTTGAGTTGAGGCCTGGCCTGGGCGC
TGGGGCCGCCGCGTGCGAATCTGGTGGCACCTTCGCGCCTGTCTCGCTGCTTTCGATAAGTCTCTAGCCATTTAAAATTTTTGATGACCT
GCTGCGACGCTTTTTTTCTGGCAAGATAGTCTTGTAAATGCGGGCCAAGATCTGCACACTGGTATTTCGGTTTTTGGGGCCGCGGGCGGC
GACGGGGCCCGTGCGTCCCAGCGCACATGTTCGGCGAGGCGGGGCCTGCGAGCGCGGCCACCGAGAATCGGACGGGGGTAGTCTCAAG
CTGGCCGGCCTGCTCTGGTGCCTGGCCTCGCGCCGCCGTGTATCGCCCCGCCCTGGGCGGCAAGGCTGGCCCGGTCGGCACCAGTTGCG
TGAGCGGAAAGATGGCCGCTTCCCGGCCCTGCTGCAGGGAGCTCAAAATGGAGGACGCGGCGCTCGGGAGAGCGGGCGGGTGAGTCAC
CCACACAAAGGAAAAGGGCCTTTCCGTCCTCAGCCGTCGCTTCATGTGACTCCACGGAGTACCGGGCGCCGTCCAGGCACCTCGATTAG
TTCTCGAGCTTTTGGAGTACGTCGTCTTTAGGTTGGGGGGAGGGGTTTTATGCGATGGAGTTTCCCCACACTGAGTGGGTGGAGACTGAA
GTTAGGCCAGCTTGGCACTTGATGTAATTCTCCTTGGAATTTGCCCTTTTTGAGTTTGGATCTTGGTTCATTCTCAAGCCTCAGACAGTGG
TTCAAAGTTTTTTTCTTCCATTTCAGGTGTCGTGACGTACGGCCACCATGACCGAGTACAAGCCCACGGTGCGCCTCGCCACCCGCGACG
ACGTCCCCAGGGCCGTACGCACCCTCGCCGCCGCGTTCGCCGACTACCCCGCCACGCGCCACACCGTCGATCCGGACCGCCACATCGAG
CGGGTCACCGAGCTGCAAGAACTCTTCCTCACGCGCGTCGGGCTCGACATCGGCAAGGTGTGGGTCGCGGACGACGGCGCCGCCGTGG
CGGTCTGGACCACGCCGGAGAGCGTCGAAGCGGGGGCGGTGTTCGCCGAGATCGGCCCGCGCATGGCCGAGTTGAGCGGTTCCCGGCT
GGCCGCGCAGCAACAGATGGAAGGCCTCCTGGCGCCGCACCGGCCCAAGGAGCCCGCGTGGTTCCTGGCCACCGTCGGAGTCTCGCCC
GACCACCAGGGCAAGGGTCTGGGCAGCGCCGTCGTGCTCCCCGGAGTGGAGGCGGCCGAGCGCGCCGGGGTGCCCGCCTTCCTGGAGA
CCTCCGCGCCCCGCAACCTCCCCTTCTACGAGCGGCTCGGCTTCACCGTCACCGCCGACGTCGAGGTGCCCGAAGGACCGCGCACCTGG
TGCATGACCCGCAAGCCCGGTGCCTGAACGCGTTAAGTCGACAATCAACCTCTGGATTACAAAATTTGTGAAAGATTGACTGGTATTCT
TAACTATGTTGCTCCTTTTACGCTATGTGGATACGCTGCTTTAATGCCTTTGTATCATGCTATTGCTTCCCGTATGGCTTTCATTTTCTCCT
CCTTGTATAAATCCTGGTTGCTGTCTCTTTATGAGGAGTTGTGGCCCGTTGTCAGGCAACGTGGCGTGGTGTGCACTGTGTTTGCTGACG
CAACCCCCACTGGTTGGGGCATTGCCACCACCTGTCAGCTCCTTTCCGGGACTTTCGCTTTCCCCCTCCCTATTGCCACGGCGGAACTCA
TCGCCGCCTGCCTTGCCCGCTGCTGGACAGGGGCTCGGCTGTTGGGCACTGACAATTCCGTGGTGTTGTCGGGGAAATCATCGTCCTTTC
CTTGGCTGCTCGCCTGTGTTGCCACCTGGATTCTGCGCGGGACGTCCTTCTGCTACGTCCCTTCGGCCCTCAATCCAGCGGACCTTCCTTC
CCGCGGCCTGCTGCCGGCTCTGCGGCCTCTTCCGCGTCTTCGCCTTCGCCCTCAGACGAGTCGGATCTCCCTTTGGGCCGCCTCCCCGCG
TCGACTTTAAGACCAATGACTTACAAGGCAGCTGTAGATCTTAGCCACTTTTTAAAAGAAAAGGGGGGACTGGAAGGGCTAATTCACTC
CCAACGAAGACAAGATCTGCTTTTTGCTTGTACTGGGTCTCTCTGGTTAGACCAGATCTGAGCCTGGGAGCTCTCTGGCTAACTAGGGAA
CCCACTGCTTAAGCCTCAATAAAGCTTGCCTTGAGTGCTTCAAGTAGTGTGTGCCCGTCTGTTGTGTGACTCTGGTAACTAGAGATCCCT
CAGACCCTTTTAGTCAGTGTGGAAAATCTCTAGCAGTACGTATAGTAGTTCATGTCATCTTATTATTCAGTATTTATAACTTGCAAAGAA
ATGAATATCAGAGAGTGAGAGGAACTTGTTTATTGCAGCTTATAATGGTTACAAATAAAGCAATAGCATCACAAATTTCACAAATAAAG

CATTTTTTTCACTGCATTCTAGTTGTGGTTTGTCCAAACTCATCAATGTATCTTATCATGTCTGGCTCTAGCTATCCCGCCCCTAACTCCGC
CCATCCCGCCCCTAACTCCGCCCAGTTCCGCCCATTCTCCGCCCCATGGCTGACTAATTTTTTTTATTTATGCAGAGGCCGAGGCCGCCTC
GGCCTCTGAGCTATTCCAGAAGTAGTGAGGAGGCTTTTTTGGAGGCCTAGGGACGTACCCAATTCGCCCTATAGTGAGTCGTATTACGC
GCGCTCACTGGCCGTCGTTTTACAACGTCGTGACTGGGAAAACCCTGGCGTTACCCAACTTAATCGCCTTGCAGCACATCCCCCTTTCGC
CAGCTGGCGTAATAGCGAAGAGGCCCGCACCGATCGCCCTTCCCAACAGTTGCGCAGCCTGAATGGCGAATGGACGCGCCCTGTAGC
GGCGCATTAAGCGCGGCGGGTGTGGTGGTTACGCGCAGCGTGACCGCTACACTTGCCAGCGCCCTAGCGCCCGCTCCTTTCGCTTTCTTC
CCTTCCTTTCTCGCCACGTTCGCCGGCTTTCCCCGTCAAGCTCTAAATCGGGGGCTCCCTTTAGGGTTCCGATTTAGTGCTTTACGGCACC
TCGACCCCAAAAAACTTGATTAGGGTGATGGTTCACGTAGTGGGCCATCGCCCTGATAGACGGTTTTTCGCCCTTTGACGTTGGAGTCCA
CGTTCTTTAATAGTGGACTCTTGTTCCAAACTGGAACAACACTCAACCCTATCTCGGTCTATTCTTTTGATTTATAAGGGATTTTGCCGAT
TTCGGCCTATTGGTTAAAAAATGAGCTGATTTAACAAAAATTTAACGCGAATTTTAACAAAATATTAACGCTTACAATTTAGGTGGCACT
TTTCGGGGAAATGTGCGCGGAACCCCTATTTGTTTATTTTTCTAAATACATTCAAATATGTATCCGCTCATGAGACAATAACCCTGATAA
ATGCTTCAATAATATTGAAAAAGGAAGAGTATGAGTATTCAACATTTCCGTGTCGCCCTTATTCCCTTTTTTGCGGCATTTTGCCTTCCTG
TTTTTGCTCACCCAGAAACGCTGGTGAAAGTAAAAGATGCTGAAGATCAGTTGGGTGCACGAGTGGGTTACATCGAACTGGATCTCAAC
AGCGGTAAGATCCTTGAGAGTTTTCGCCCCGAAGAACGTTTTCCAATGATGAGCACTTTTAAAGTTCTGCTATGTGGCGCGGTATTATCC
CGTATTGACGCCGGGCAAGAGCAACTCGGTCGCCGCATACACTATTCTCAGAATGACTTGGTTGAGTACTCACCAGTCACAGAAAAGCA
TCTTACGGATGGCATGACAGTAAGAGAATTATGCAGTGCTGCCATAACCATGAGTGATAACACTGCGGCCAACTTACTTCTGACAACGA
TCGGAGGACCGAAGGAGCTAACCGCTTTTTTGCACAACATGGGGGATCATGTAACTCGCCTTGATCGTTGGGAACCGGAGCTGAATGAA
GCCATACCAAACGACGAGCGTGACACCACGATGCCTGTAGCAATGGCAACAACGTTGCGCAAACTATTAACTGGCGAACTACTTACTCT
AGCTTCCCGGCAACAATTAATAGACTGGATGGAGGCGGATAAAGTTGCAGGACCACTTCTGCGCTCGGCCCTTCCGGCTGGCTGGTTTA
TTGCTGATAAATCTGGAGCCGGTGAGCGTGGGTCTCGCGGTATCATTGCAGCACTGGGGCCAGATGGTAAGCCCTCCCGTATCGTAGTT
ATCTACACGACGGGGAGTCAGGCAACTATGGATGAACGAAATAGACAGATCGCTGAGATAGGTGCCTCACTGATTAAGCATTGGTAAA
CTGTCAGACCAAGTTTACTCATATATACTTTAGATTGATTTAAAACTTCATTTTTAATTTAAAAGGATCTAGGTGAAGATCCTTTTTGATA
ATCTCATGACCAAAATCCCTTAACGTGAGTTTTCGTTCCACTGAGCGTCAGACCCCGTAGAAAAGATCAAAGGATCTTCTTGAGATCCTT
TTTTTCTGCGCGTAATCTGCTGCTTGCAAACAAAAAAACCACCGCTACCAGCGGTGGTTTGTTTGCCGGATCAAGAGCTACCAACTCTTT
TTCCGAAGGTAACTGGCTTCAGCAGAGCGCAGATACCAAATACTGTTCTTCTAGTGTAGCCGTAGTTAGGCCACCACTTCAAGAACTCT
GTAGCACCGCCTACATACCTCGCTCTGCTAATCCTGTTACCAGTGGCTGCTGCCAGTGGCGATAAGTCGTGTCTTACCGGGTTGGACTCA
AGACGATAGTTACCGGATAAGGCGCAGCGGTCGGGCTGAACGGGGGGTTCGTGCACACAGCCCAGCTTGGAGCGAACGACCTACACCG
AACTGAGATACCTACAGCGTGAGCTATGAGAAAGCGCCACGCTTCCCGAAGGGAGAAAGGCGGACAGGTATCCGGTAAGCGGCAGGG
TCGGAACAGGAGAGCGCACGAGGGAGCTTCCAGGGGGAAACGCCTGGTATCTTTATAGTCCTGTCGGGTTTCGCCACCTCTGACTTGAG
CGTCGATTTTTGTGATGCTCGTCAGGGGGGCGGAGCCTATGGAAAAACGCCAGCAACGCGGCCTTTTTACGGTTCCTGGCCTTTTGCTGG
CCTTTTGCTCACATGTTCTTTCCTGCGTTATCCCCTGATTCTGTGGATAACCGTATTACCGCCTTTGAGTGAGCTGATACCGCTCGCCGCA
GCCGAACGACCGAGCGCAGCGAGTCAGTGAGCGAGGAAGCGGAAGAGCGCCCAATACGCAAACCGCCTCTCCCCGCGCGTTGGCCGAT
TCATTAATGCAGCTGGCACGACAGGTTTCCCGACTGGAAAGCGGGCAGTGAGCGCAACGCAATTAATGTGAGTTAGCTCACTCATTAGG
CACCCCAGGCTTTACACTTTATGCTTCCGGCTCGTATGTTGTGTGGAATTGTGAGCGGATAACAATTTCACACAGGAAACAGCTATGACC
ATGATTACGCCAAGCGCGCAATTAACCCTCACTAAAGGGAACAAAAGCTGGAGCTGCAAGCTTAATGTAGTCTTATGCAATACTCTTGT
AGTCTTGCAACATGGTAACGATGAGTTAGCAACATGCCTTACAAGGAGAGAAAAAGCACCGTGCATGCCGATTGGTGGAAGTAAGGTG
GTACGATCGTGCCTTATTAGGAAGGCAACAGACGGGTCTGACATGGATTGGACGAACCACTGAATTGCCGCATTGCAGAGATATTGTAT
TTAAGTGCCTAGCTCGATACATAAACGGGTCTCTCTGGTTAGACCAGATCTGAGCCTGGGAGCTCTCTGGCTAACTAGGGAACCCACTG
CTTAAGCCTCAATAAAGCTTGCCTTGAGTGCTTCAAGTAGTGTGTGCCCGTCTGTTGTGTGACTCTGGTAACTAGAGATCCCTCAGACCC
TTTTAGTCAGTGTGGAAAATCTCTAGCAGTGGCGCCCGAACAGGGACTTGAAAGCGAAAGGGAAACCAGAGGAGCTCTCTCGACGCAG
GACTCGGCTTGCTGAAGCGCGCACGGCAAGAGGCGAGGGGCGGCGACTGGTGAGTACGCCAAAAATTTTGACTAGCGGAGGCTAGAAG
GAGAGAGATGGGTGCGAGAGCGTCAGTATTAAGCGGGGGAGAATTAGATCGCGATGGGAAAAAATTCGGTTAAGGCCAGGGGGAAAG
AAAAAATATAAATTAAAACATATAGTATGGGCAAGCAGGGAGCTAGAACGATTCGCAGTTAATCCTGGCCTGTTAGAAACATCAGAAG
GCTGTAGACAAATACTGGGACAGCTACAACCATCCCTTCAGACAGGATCAGAAGAACTTAGATCATTATATAATACAGTAGCAACCCTC
TATTGTGTGCATCAAAGGATAGAGATAAAAGACACCAAGGAAGCTTTAGACAAGATAGAGGAAGAGCAAAACAAAAGTAAGACCACC
GCACAGCAAGCGGCCGCTGATCTTCAGACCTGGAGGAGGAGATATGAGGGACAATTGGAGAAGTGAATTATATAAATATAAAGTAGTA
AAAATTGAACCATTAGGAGTAGCACCCACCAAGGCAAAGAGAAGAGTGGTGCAGAGAGAAAAAAGAGCAGTGGGAATAGGAGCTTTG
TTCCTTGGGTTCTTGGGAGCAGCAGGAAGCACTATGGGCGCAGCGTCAATGACGCTGACGGTACAGGCCAGACAATTATTGTCTGGTAT
AGTGCAGCAGCAGAACAATTTGCTGAGGGCTATTGAGGCGCAACAGCATCTGTTGCAACTCACAGTCTGGGGCATCAAGCAGCTCCAG
GCAAGAATCCTGGCTGTGGAAAGATACCTAAAGGATCAACAGCTCCTGGGGATTTGGGGTTGCTCTGGAAAACTCATTTGCACCACTGC
TGTGCCTTGGAATGCTAGTTGGAGTAATAAATCTCTGGAACAGATTTGGAATCACACGACCTGGATGGAGTGGGACAGAGAAATTAAC
AATTACACAAGCTTAATACACTCCTTAATTGAAGAATCGCAAAACCAGCAAGAAAAGAATGAACAAGAATTATTGGAATTAGATAAAT
GGGCAAGTTTGTGGAATTGGTTTAACA

pB-pCMV-PEmax-P2A-hMLH1dn vector

Plasmid backbone

CMV enhancer and promoter

SV40 NLS

SpCas9 (R221K N394K H840A)

c-myc NLS

EF-1-alpha core promoter

BSD

ACTCTTCCTTTTTCAATATTATTGAAGCATTTATCAGGGTTATTGTCTCATGAGCGGATACATATTTGAATGTATTTAGAAAAATAAACA
AATAGGGGTTCCGCGCACATTTCCCCGAAAAGTGCCACCTAAATTGTAAGCGTTAATATTTTGTTAAAATTCGCGTTAAATTTTTGTTAA
ATCAGCTCATTTTTTAACCAATAGGCCGAAATCGGCAAAATCCCTTATAAATCAAAAGAATAGACCGAGATAGGGTTGAGTGTTGTTCC
AGTTTGGAACAAGAGTCCACTATTAAAGAACGTGGACTCCAACGTCAAAGGGCGAAAAACCGTCTATCAGGGCGATGGCCCACTACGT
GAACCATCACCCTAATCAAGTTTTTTGGGGTCGAGGTGCCGTAAAGCACTAAATCGGAACCCTAAAGGGAGCCCCCGATTTAGAGCTTG
ACGGGGAAAGCCGGCGAACGTGGCGAGAAAGGAAGGGAAGAAAGCGAAAGGAGCGGGCGCTAGGGCGCTGGCAAGTGTAGCGGTCA
CGCTGCGCGTAACCACCACACCCGCCGCGCTTAATGCGCCGCTACAGGGCGCGTCCCATTCGCCATTCAGGCTGCGCAACTGTTGGGAA
GGGCGATCGGTGCGGGCCTCTTCGCTATTACGCCAGCTGGCGAAAGGGGGATGTGCTGCAAGGCGATTAAGTTGGGTAACGCCAGGGT
TTTCCCAGTCACGACGTTGTAAAACGACGGCCAGTGAGCGCGCCTCGTTCATTCACGTTTTTGAACCCGTGGAGGACGGGCAGACTCGC
GGTGCAAATGTGTTTTACAGCGTGATGGAGCAGATGAAGATGCTCGACACGCTGCAGAACACGCAGCTAGATTAACCCTAGAAAGATA
ATCATATTGTGACGTACGTTAAAGATAATCATGTGTAAAATTGACGCATGTGTTTTATCGGTCTGTATATCGAGGTTTATTTATTAATTTG
AATAGATATTAAGTTTTATTATATTTACACTTACATACTAATAATAAATTCAACAAACAATTTATTTATGTTTATTTATTTATTAAAAAAA
ACAAAAACTCAAAATTTCTTCTATAAAGTAACAAAACTTTTATGAGGGACAGCCCCCCCCCAAAGCCCCCAGGGATGTAATTACGTCCC
TCCCCCGCTAGGGGGCAGCAGCGAGCCGCCCGGGGCTCCGCTCCGGTCCGGCGCTCCCCCCGCATCCCCGAGCCGGCAGCGTGCGGGG
ACAGCCCGGGCACGGGGAAGGTGGCACGGGATCGCTTTCCTCTGAACGCTTCTCGCTGCTCTTTGAGCCTGCAGACACCTGGGGGGATA
CGGGGAAAAGGCCTCCACGGCCGACATTGATTATTGACTAGTTATTAATAGTAATCAATTACGGGGTCATTAGTTCATAGCCCATATAT
GGAGTTCCGCGTTACATAACTTACGGTAAATGGCCCGCCTGGCTGACCGCCCAACGACCCCCGCCCATTGACGTCAATAATGACGTATG
TTCCCATAGTAACGCCAATAGGGACTTTCCATTGACGTCAATGGGTGGAGTATTTACGGTAAACTGCCCACTTGGCAGTACATCAAGTG
TATCATATGCCAAGTACGCCCCCTATTGACGTCAATGACGGTAAATGGCCCGCCTGGCATTATGCCCAGTACATGACCTTATGGGACTTT
CCTACTTGGCAGTACATCTACGTATTAGTCATCGCTATTACCATGGTGATGCGGTTTTGGCAGTACATCAATGGGCGTGGATAGCGGTTT
GACTCACGGGGATTTCCAAGTCTCCACCCCATTGACGTCAATGGGAGTTTGTTTTGGCACCAAAATCAACGGGACTTTCCAAAATGTCGT
AACAACTCCGCCCCATTGACGCAAATGGGCGGTAGGCGTGTACGGTGGGAGGTCTATATAAGCAGAGCTGGTTTAGTGAACCGTCAGA
TCCGCTAGAGATCCGCGGCCGCTAATACGACTCACTATAGGGAGAGCCGCCACCATGAAACGGACAGCCGACGGAAGCGAGTTCGAGT
CACCAAAGAAGAAGCGGAAAGTCGACAAGAAGTACAGCATCGGCCTGGACATCGGCACCAACTCTGTGGGCTGGGCCGTGATCACCGA
CGAGTACAAGGTGCCCAGCAAGAAATTCAAGGTGCTGGGCAACACCGACCGGCACAGCATCAAGAAGAACCTGATCGGAGCCCTGCTG
TTCGACAGCGGCGAAACAGCCGAGGCCACCCGGCTGAAGAGAACCGCCAGAAGAAGATACACCAGACGGAAGAACCGGATCTGCTAT
CTGCAAGAGATCTTCAGCAACGAGATGGCCAAGGTGGACGACAGCTTCTTCCACAGACTGGAAGAGTCCTTCCTGGTGGAAGAGGATA
AGAAGCACGAGCGGCACCCCATCTTCGGCAACATCGTGGACGAGGTGGCCTACCACGAGAAGTACCCCACCATCTACCACCTGAGAAA
GAAACTGGTGGACAGCACCGACAAGGCCGACCTGCGGCTGATCTATCTGGCCCTGGCCCACATGATCAAGTTCCGGGGCCACTTCCTGA
TCGAGGGCGACCTGAACCCCGACAACAGCGACGTGGACAAGCTGTTCATCCAGCTGGTGCAGACCTACAACCAGCTGTTCGAGGAAAA
CCCCATCAACGCCAGCGGCGTGGACGCCAAGGCCATCCTGTCTGCCAGACTGAGCAAGAGCAGAAAGCTGGAAAATCTGATCGCCCAG
CTGCCCGGCGAGAAGAAGAATGGCCTGTTCGGAAACCTGATTGCCCTGAGCCTGGGCCTGACCCCCAACTTCAAGAGCAACTTCGACCT
GGCCGAGGATGCCAAACTGCAGCTGAGCAAGGACACCTACGACGACGACCTGGACAACCTGCTGGCCCAGATCGGCGACCAGTACGCC
GACCTGTTTCTGGCCGCCAAGAACCTGTCCGACGCCATCCTGCTGAGCGACATCCTGAGAGTGAACACCGAGATCACCAAGGCCCCCCT
GAGCGCCTCTATGATCAAGAGATACGACGAGCACCACCAGGACCTGACCCTGCTGAAAGCTCTCGTGCGGCAGCAGCTGCCTGAGAAG
TACAAAGAGATTTTCTTCGACCAGAGCAAGAACGGCTACGCCGGCTACATTGACGGCGGAGCCAGCCAGGAAGAGTTCTACAAGTTCA
TCAAGCCCATCCTGGAAAAGATGGACGGCACCGAGGAACTGCTCGTGAAGCTGAAGAGAGAGGACCTGCTGCGGAAGCAGCGGACCTT
CGACAACGGCAGCATCCCCCACCAGATCCACCTGGGAGAGCTGCACGCCATTCTGCGGCGGCAGGAAGATTTTTACCCATTCCTGAAGG
ACAACCGGGAAAAGATCGAGAAGATCCTGACCTTCCGCATCCCCTACTACGTGGGCCCTCTGGCCAGGGGAAACAGCAGATTCGCCTG
GATGACCAGAAAGAGCGAGGAAACCATCACCCCCTGGAACTTCGAGGAAGTGGTGGACAAGGGCGCTTCCGCCCAGAGCTTCATCGAG
CGGATGACCAACTTCGATAAGAACCTGCCCAACGAGAAGGTGCTGCCCAAGCACAGCCTGCTGTACGAGTACTTCACCGTGTATAACGA
GCTGACCAAAGTGAAATACGTGACCGAGGGAATGAGAAAGCCCGCCTTCCTGAGCGGCGAGCAGAAAAAGGCCATCGTGGACCTGCTG
TTCAAGACCAACCGGAAAGTGACCGTGAAGCAGCTGAAAGAGGACTACTTCAAGAAAATCGAGTGCTTCGACTCCGTGGAAATCTCCG
GCGTGGAAGATCGGTTCAACGCCTCCCTGGGCACATACCACGATCTGCTGAAAATTATCAAGGACAAGGACTTCCTGGACAATGAGGA
AAACGAGGACATTCTGGAAGATATCGTGCTGACCCTGACACTGTTTGAGGACAGAGAGATGATCGAGGAACGGCTGAAAACCTATGCC
CACCTGTTCGACGACAAAGTGATGAAGCAGCTGAAGCGGCGGAGATACACCGGCTGGGGCAGGCTGAGCCGGAAGCTGATCAACGGC
ATCCGGGACAAGCAGTCCGGCAAGACAATCCTGGATTTCCTGAAGTCCGACGGCTTCGCCAACAGAAACTTCATGCAGCTGATCCACGA
CGACAGCCTGACCTTTAAAGAGGACATCCAGAAAGCCCAGGTGTCCGGCCAGGGCGATAGCCTGCACGAGCACATTGCCAATCTGGCC
GGCAGCCCCGCCATTAAGAAGGGCATCCTGCAGACAGTGAAGGTGGTGGACGAGCTCGTGAAAGTGATGGGCCGGCACAAGCCCGAG
AACATCGTGATCGAAATGGCCAGAGAGAACCAGACCACCCAGAAGGGACAGAAGAACAGCCGCGAGAGAATGAAGCGGATCGAAGA
GGGCATCAAAGAGCTGGGCAGCCAGATCCTGAAAGAACACCCCGTGGAAAACACCCAGCTGCAGAACGAGAAGCTGTACCTGTACTAC
CTGCAGAATGGGCGGGATATGTACGTGGACCAGGAACTGGACATCAACCGGCTGTCCGACTACGATGTGGACGCTATCGTGCCTCAGA
GCTTTCTGAAGGACGACTCCATCGACAACAAGGTGCTGACCAGAAGCGACAAGAACCGGGGCAAGAGCGACAACGTGCCCTCCGAAGA
GGTCGTGAAGAAGATGAAGAACTACTGGCGGCAGCTGCTGAACGCCAAGCTGATTACCCAGAGAAAGTTCGACAATCTGACCAAGGCC
GAGAGAGGCGGCCTGAGCGAACTGGATAAGGCCGGCTTCATCAAGAGACAGCTGGTGGAAACCCGGCAGATCACAAAGCACGTGGCA
CAGATCCTGGACTCCCGGATGAACACTAAGTACGACGAGAATGACAAGCTGATCCGGGAAGTGAAAGTGATCACCCTGAAGTCCAAGC
TGGTGTCCGATTTCCGGAAGGATTTCCAGTTTTACAAAGTGCGCGAGATCAACAACTACCACCACGCCCACGACGCCTACCTGAACGCC
GTCGTGGGAACCGCCCTGATCAAAAAGTACCCTAAGCTGGAAAGCGAGTTCGTGTACGGCGACTACAAGGTGTACGACGTGCGGAAGA
TGATCGCCAAGAGCGAGCAGGAAATCGGCAAGGCTACCGCCAAGTACTTCTTCTACAGCAACATCATGAACTTTTTCAAGACCGAGATT
ACCCTGGCCAACGGCGAGATCCGGAAGCGGCCTCTGATCGAGACAAACGGCGAAACCGGGGAGATCGTGTGGGATAAGGGCCGGGAT
TTTGCCACCGTGCGGAAAGTGCTGAGCATGCCCCAAGTGAATATCGTGAAAAAGACCGAGGTGCAGACAGGCGGCTTCAGCAAAGAGT
CTATCCTGCCCAAGAGGAACAGCGATAAGCTGATCGCCAGAAAGAAGGACTGGGACCCTAAGAAGTACGGCGGCTTCGACAGCCCCAC
CGTGGCCTATTCTGTGCTGGTGGTGGCCAAAGTGGAAAAGGGCAAGTCCAAGAAACTGAAGAGTGTGAAAGAGCTGCTGGGGATCACC
ATCATGGAAAGAAGCAGCTTCGAGAAGAATCCCATCGACTTTCTGGAAGCCAAGGGCTACAAAGAAGTGAAAAAGGACCTGATCATCA
AGCTGCCTAAGTACTCCCTGTTCGAGCTGGAAAACGGCCGGAAGAGAATGCTGGCCTCTGCCGGCGAACTGCAGAAGGGAAACGAACT
GGCCCTGCCCTCCAAATATGTGAACTTCCTGTACCTGGCCAGCCACTATGAGAAGCTGAAGGGCTCCCCCGAGGATAATGAGCAGAAAC
AGCTGTTTGTGGAACAGCACAAGCACTACCTGGACGAGATCATCGAGCAGATCAGCGAGTTCTCCAAGAGAGTGATCCTGGCCGACGC
TAATCTGGACAAAGTGCTGTCCGCCTACAACAAGCACCGGGATAAGCCCATCAGAGAGCAGGCCGAGAATATCATCCACCTGTTTACCC
TGACCAATCTGGGAGCCCCTGCCGCCTTCAAGTACTTTGACACCACCATCGACCGGAAGAGGTACACCAGCACCAAAGAGGTGCTGGA
CGCCACCCTGATCCACCAGAGCATCACCGGCCTGTACGAGACACGGATCGACCTGTCTCAGCTGGGAGGTGACTCCGGCGGAAGCTCTG
GTGGCAGCAAGCGGACCGCCGACGGCTCTGAATTCGAGAGCCCTAAGAAGAAAAGAAAGGTGAGCGGAGGCTCTAGCGGCGGAAGCA

CCCTGAACATTGAAGACGAGTATAGACTGCATGAAACAAGCAAGGAACCCGACGTGTCCCTGGGCTCCACCTGGCTGTCCGACTTTCCC
CAGGCCTGGGCCGAGACAGGAGGAATGGGCCTGGCCGTGCGGCAGGCACCCCTGATCATCCCTCTGAAGGCCACCTCTACACCCGTGA
GCATCAAGCAGTACCCTATGTCTCAGGAGGCCAGACTGGGCATCAAGCCTCACATCCAGAGGCTGCTGGACCAGGGCATCCTGGTGCCA
TGCCAGAGCCCCTGGAACACACCACTGCTGCCCGTGAAGAAGCCAGGCACCAATGACTATAGACCCGTGCAGGATCTGAGAGAGGTGA
ACAAGAGGGTGGAGGATATCCACCCCACCGTGCCCAACCCTTACAATCTGCTGTCCGGCCTGCCCCCTTCTCACCAGTGGTATACAGTG
CTGGACCTGAAGGATGCCTTCTTTTGTCTGAGACTGCACCCTACCAGCCAGCCACTGTTCGCCTTTGAGTGGAGGGACCCTGAGATGGG
CATCTCTGGCCAGCTGACCTGGACACGCCTGCCTCAGGGCTTCAAGAATAGCCCAACACTGTTTAACGAGGCCCTGCACCGCGACCTGG
CAGATTTCCGGATCCAGCACCCAGATCTGATCCTGCTGCAGTACGTGGACGATCTGCTGCTGGCCGCCACCAGCGAGCTGGATTGCCAG
CAGGGAACACGCGCCCTGCTGCAGACCCTGGGAAACCTGGGATATAGGGCATCCGCCAAGAAGGCCCAGATCTGTCAGAAGCAGGTGA
AGTACCTGGGCTATCTGCTGAAGGAGGGCCAGAGATGGCTGACAGAGGCCAGGAAGGAGACAGTGATGGGCCAGCCAACACCCAAGA
CCCCAAGACAGCTGAGGGAGTTCCTGGGCAAAGCAGGATTTTGCAGGCTGTTCATCCCAGGATTCGCAGAGATGGCAGCACCTCTGTAC
CCACTGACCAAGCCGGGCACCCTGTTTAATTGGGGCCCTGACCAGCAGAAGGCCTATCAGGAGATCAAGCAGGCCCTGCTGACAGCAC
CAGCCCTGGGCCTGCCAGACCTGACCAAGCCTTTCGAGCTGTTTGTGGATGAGAAGCAGGGCTACGCCAAGGGCGTGCTGACCCAGAA
GCTGGGACCATGGAGACGGCCCGTGGCCTATCTGTCCAAGAAGCTGGACCCAGTGGCAGCAGGATGGCCACCATGCCTGAGGATGGTG
GCAGCAATCGCCGTGCTGACAAAGGATGCCGGCAAGCTGACCATGGGACAGCCACTGGTCATCCTGGCACCACACGCAGTGGAGGCCC
TGGTGAAGCAGCCTCCAGATCGCTGGCTGTCTAACGCCCGGATGACACACTACCAGGCCCTGCTGCTGGACACCGATCGCGTGCAGTTT
GGCCCTGTGGTGGCCCTGAATCCAGCCACCCTGCTGCCTCTGCCAGAGGAGGGCCTGCAGCACAACTGTCTGGACATCCTGGCAGAGGC
ACACGGAACAAGGCCAGACCTGACCGATCAGCCCCTGCCTGACGCCGATCACACATGGTATACCGATGGAAGCTCCCTGCTGCAGGAG
GGCCAGAGGAAGGCAGGAGCAGCAGTGACCACAGAGACAGAAGTGATCTGGGCCAAGGCCCTGCCAGCAGGCACATCCGCCCAGCGG
GCCGAGCTGATCGCCCTGACCCAGGCCCTGAAGATGGCCGAGGGCAAGAAGCTGAACGTGTACACAGACTCCAGATATGCCTTCGCCA
CCGCACACATCCACGGAGAGATCTACAGGCGCCGGGGCTGGCTGACCTCTGAGGGCAAGGAGATCAAGAACAAGGATGAGATCCTGGC
CCTGCTGAAGGCCCTGTTTCTGCCCAAGCGGCTGAGCATCATCCACTGTCCTGGACACCAGAAGGGACACTCCGCCGAGGCAAGGGGCA
ATCGGATGGCCGACCAGGCCGCCAGAAAGGCTGCTATTACTGAAACTCCCGACACTTCCACTCTGCTGATTGAAAACTCCTCCCCTTCTG
GCGGCTCAAAAAGAACCGCCGACGGCAGCGAATTCGAGTCTCCCAAGAAGAAGAGGAAAGTCGGCTCTGGCCCTGCCGCTAAGAGAGT
GAAGCTGGACGGAAGCGGAGCTACTAACTTCAGCCTGCTGAAGCAGGCTGGAGACGTGGAGGAGAACCCTGGACCTAGCTTCGTTGCT
GGAGTCATCCGGAGACTGGACGAGACAGTGGTGAACAGAATTGCCGCCGGCGAGGTGATCCAGAGACCTGCCAATGCAATAAAGGAG
ATGATCGAGAACTGTCTGGACGCCAAGTCCACAAGCATTCAGGTGATCGTGAAGGAGGGCGGACTGAAGCTGATCCAGATCCAAGACA
ACGGCACAGGCATCAGAAAGGAAGATCTGGACATCGTGTGTGAACGGTTCACCACATCTAAGCTGCAGTCTTTTGAGGATCTGGCCTCT
ATCAGTACCTACGGCTTCAGAGGCGAGGCCCTGGCCAGCATCAGCCACGTGGCCCATGTGACCATCACCACCAAAACCGCCGACGGCA
AATGCGCTTATCGCGCTAGCTACAGCGACGGCAAGCTGAAAGCCCCGCCAAAGCCTTGCGCCGGCAACCAGGGTACACAGATAACAGT
GGAGGATCTGTTCTACAACATCGCCACCCGGAGAAAGGCCCTGAAAAATCCCAGCGAGGAGTACGGCAAGATCCTGGAAGTCGTCGGC
AGATACTCCGTGCACAACGCCGGAATCAGCTTTAGCGTAAAGAAGCAGGGAGAAACCGTGGCCGATGTGCGCACCCTGCCAAATGCCA
GCACCGTGGATAACATCAGAAGCATTTTCGGAAATGCCGTGTCCAGAGAACTGATCGAGATCGGCTGCGAAGATAAGACCCTGGCTTTT
AAGATGAACGGCTACATCAGCAACGCCAATTACTCTGTGAAGAAGTGCATCTTTCTTCTGTTCATCAACCACAGACTGGTGGAAAGCAC
CAGCCTGCGGAAAGCCATCGAGACAGTGTACGCCGCCTACCTGCCTAAGAACACCCACCCCTTCCTGTACCTGAGCCTCGAGATCAGCC
CTCAGAACGTGGACGTCAATGTGCATCCTACAAAGCACGAGGTGCACTTCCTGCACGAGGAAAGCATCCTGGAAAGAGTGCAGCAGCA
CATTGAGAGCAAGCTGCTGGGCTCTAACAGCAGCAGAATGTACTTCACACAGACCCTGCTGCCTGGCCTGGCCGGCCCCTCAGGCGAAA
TGGTTAAGTCCACAACCTCTCTGACCTCATCCAGCACCAGCGGTTCTTCCGATAAGGTGTACGCCCACCAGATGGTGCGGACCGACTCTC
GGGAGCAGAAGCTGGACGCCTTTCTGCAACCTCTGAGCAAACCTCTGAGCTCTCAGCCTCAGGCCATCGTGACCGAGGACAAGACAGA
TATCTCCTCCGGCCGTGCCAGACAGCAGGACGAAGAAATGCTCGAGCTGCCAGCTCCTGCCGAGGTGGCCGCCAAGAACCAGAGCCTG
GAGGGAGATACCACAAAGGGCACCAGCGAAATGAGCGAGAAGCGGGGCCCTACCTCCAGCAACCCCAGAAAACGGCACCGGGAGGAC
AGCGACGTGGAAATGGTGGAGGACGACAGCAGAAAGGAAATGACAGCCGCTTGTACCCCTAGAAGAAGAATCATCAACCTGACCTCCG
TGCTGAGCCTGCAGGAGGAGATCAACGAGCAGGGCCACGAGGTGCTGAGAGAGATGCTGCACAATCACAGCTTCGTGGGCTGCGTGAA
CCCTCAATGGGCCCTGGCTCAGCATCAAACAAAGCTGTACCTGCTGAACACCACCAAGCTGAGCGAAGAGCTGTTCTACCAGATCCTCA
TCTACGACTTCGCCAACTTCGGCGTGCTACGCCTGAGCGAGCCCGCCCCTCTGTTTGACCTGGCCATGCTGGCTCTGGATAGCCCAGAAA
GCGGCTGGACAGAAGAGGACGGACCTAAAGAGGGGCTGGCTGAATACATCGTGGAGTTCCTGAAGAAAAAGGCCGAGATGCTGGCCG
ACTACTTTTCTCTGGAAATCGACGAGGAAGGCAACCTGATCGGCCTGCCTCTGCTGATCGATAACTACGTGCCTCCCCTGGAAGGCCTG
CCCATCTTCATCCTGAGACTGGCTACAGAGGTGAACTGGGACGAGGAAAAGGAATGCTTCGAGTCTCTGAGCAAGGAGTGCGCCATGTT
CTATAGCATCAGAAAACAGTACATCTCTGAAGAGAGCACTCTGTCTGGCCAGCAGAGTGAAGTGCCCGGAAGCATCCCCAACAGCTGG
AAGTGGACCGTGGAACACATCGTGTACAAGGCCCTGCGGAGCCACATTCTCCCTCCTAAGCACTTCACCGAGGACGGCAACATCCTGCA
GCTGGCCAACCTGCCCGACCTTTATAAGGTTTTCTAACCGGTCATCATCACCATCACCATTGAGTTTAAACGGGGGAGGCTAACTGAAA
CACGGAAGGAGACAATACCGGAAGGAACCCGCGCTATGACGGCAATAAAAAGACAGAATAAAACGCACGGGTGTTGGGTCGTTTGTTC
ATAAACGCGGGGTTCGGTCCCAGGGCTGGCACTCTGTCGATACCCCACCGAGACCCCATTGGGGCCAATACGCCCGCGTTTCTTCCTTTT
CCCCACCCCACCCCCCAAGTTCGGGTGAAGGCCCAGGGCTCGCAGCCAACGTCGGGGCGGCAGGCCCTGCCATAGCAGATCTGCGAAA
TCGGATCCGCGGCCGCACTGATTCTAGCTGCAGAGGCTCCGGTGCCCGTCAGTGGGCAGAGCGCACATCGCCCACAGTCCCCGAGAAGT
TGGGGGGAGGGGTCGGCAATTGAACCGGTGCCTAGAGAAGGTGGCGCGGGGTAAACTGGGAAAGTGATGTCGTGTACTGGCTCCGCCT
TTTTCCCGAGGGTGGGGGAGAACCGTATATAAGTGCAGTAGTCGCCGTGAACGTTCTTTTTCGCAACGGGTTTGCCGCCAGAACACAGG
ATCCTCGAGCCACCATGGCCAAGCCTTTGTCTCAAGAAGAATCCACCCTCATTGAAAGAGCAACGGCTACAATCAACAGCATCCCCATC
TCTGAAGACTACAGCGTCGCCAGCGCAGCTCTCTCTAGCGACGGCCGCATCTTCACTGGTGTCAATGTATATCATTTTACTGGGGACCT
TGTGCAGAACTCGTGGTGCTGGGCACTGCTGCTGCTGCGGCAGCTGGCAACCTGACTTGTATCGTCGCGATCGGAAATGAGAACAGGGG
CATCTTGAGCCCCTGCGGACGGTGCCGACAGGTGCTTCTCGATCTGCATCCTGGGATCAAAGCCATAGTGAAGGACAGTGATGGACAGC
CGACGGCAGTTGGGATTCGTGAATTGCTGCCCTCTGGTTATGTGTGGGAGGGCTAAGTTAACTTGTTTATTGCAGCTTATAATGGTTACA
AATAAAGCAATAGCATCACAAATTTCACAAATAAAGCATTTTTTTCACTGCATTCTAGTTGTGGTTTGTCCAAACTCATCAATGTATCTT
ATCATGTCTGGAATTGACTCAAATGATGTCAATTAGTCTATCAGAAGCTCATCTGGTCTCCCTTCCGGGGGACAAGACATCCCTGTTTAA
TATTTAAACAGCAGTGTTCCCAAACTGGGTTCTTATATCCCTTGCTCTGGTCAACCAGGTTGCAGGGTTTCCTGTCCTCACAGGAACGAA
GTCCCTAAAGAAACAGTGGCAGCCAGGTTTAGCCCCGGAATTGACTGGATTCCTTTTTTAGGGCCCATTGGTATGGCTTTTTCCCCGTAT
CCCCCCAGGTGTCTGCAGGCTCAAAGAGCAGCGAGAAGCGTTCAGAGGAAAGCGATCCCGTGCCACCTTCCCCGTGCCCGGCTGTCCC
CGCACGCTGCCGGCTCGGGGATGCGGGGGGAGCGCCGGACCGGAGCGGAGCCCCGGGCGGCTCGCTGCTGCCCCCTAGCGGGGGAGG
GACGTAATTACATCCCTGGGGGCTTTGGGGGGGGGCTGTCCCTGATATCTATAACAAGAAAATATATATATAATAAGTTATCACGTAAG
TAGAACATGAAATAACAATATAATTATCGTATGAGTTAAATCTTAAAAGTCACGTAAAAGATAATCATGCGTCATTTTGACTCACGCGG
TCGTTATAGTTCAAAATCAGTGACACTTACCGCATTGACAAGCACGCCTCACGGGAGCTCCAAGCGGCGACTGAGATGTCCTAAATGCA
CAGCGACGGATTCGCGCTATTTAGAAAGAGAGAGCAATATTTCAAGAATGCATGCGTCAATTTTACGCAGACTATCTTTCTAGGGTTAA

TCTAGCTGCATCAGGATCATATCGTCGGGTCTTTTTTCCGGCTCAGTCATCGCCCAAGCTGGCGCTATCTGGGCATCGGGGAGGAAGAA
GCCCGTGCCTTTTCCCGCGAGGTTGAAGCGGCATGGAAAGAGTTTGCCGAGGATGACTGCTGCTGCATTGACGTTGAGCGAAAACGCAC
GTTTACCATGATGATTCGGGAAGGTGTGGCCATGCACGCCTTTAACGGTGAACTGTTCGTTCAGGCCACCTGGGATACCAGTTCGTCGC
GGCTTTTCCGGACACAGTTCCGGATGGTCAGCCCGAAGCGCATCAGCAACCCGAACAATACCGGCGACAGCCGGAACTGCCGTGCCGG
TGTGCAGATTAATGACAGCGGTGCGGCGCTGGGATATTACGTCAGCGAGGACGGGTATCCTGGCTGGATGCCGCAGAAATGGACATGG
ATACCCCGTGAGTTACCCGGCGGGCGCGCTTGGCGTAATCATGGTCATAGCTGTTTCCTGTGTGAAATTGTTATCCGCTCACAATTCCAC
ACAACATACGAGCCGGAAGCATAAAGTGTAAAGCCTGGGGTGCCTAATGAGTGAGCTAACTCACATTAATTGCGTTGCGCTCACTGCCC
GCTTTCCAGTCGGGAAACCTGTCGTGCCAGCTGCATTAATGAATCGGCCAACGCGCGGGGAGAGGCGGTTTGCGTATTGGGCGCTCTTC
CGCTTCCTCGCTCACTGACTCGCTGCGCTCGGTCGTTCGGCTGCGGCGAGCGGTATCAGCTCACTCAAAGGCGGTAATACGGTTATCCAC
AGAATCAGGGGATAACGCAGGAAAGAACATGTGAGCAAAAGGCCAGCAAAAGGCCAGGAACCGTAAAAAGGCCGCGTTGCTGGCGTT
TTTCCATAGGCTCCGCCCCCCTGACGAGCATCACAAAAATCGACGCTCAAGTCAGAGGTGGCGAAACCCGACAGGACTATAAAGATAC
CAGGCGTTTCCCCCTGGAAGCTCCCTCGTGCGCTCTCCTGTTCCGACCCTGCCGCTTACCGGATACCTGTCCGCCTTTCTCCCTTCGGGAA
GCGTGGCGCTTTCTCATAGCTCACGCTGTAGGTATCTCAGTTCGGTGTAGGTCGTTCGCTCCAAGCTGGGCTGTGTGCACGAACCCCCCG
TTCAGCCCGACCGCTGCGCCTTATCCGGTAACTATCGTCTTGAGTCCAACCCGGTAAGACACGACTTATCGCCACTGGCAGCAGCCACT
GGTAACAGGATTAGCAGAGCGAGGTATGTAGGCGGTGCTACAGAGTTCTTGAAGTGGTGGCCTAACTACGGCTACACTAGAAGAACAG
TATTTGGTATCTGCGCTCTGCTGAAGCCAGTTACCTTCGGAAAAAGAGTTGGTAGCTCTTGATCCGGCAAACAAACCACCGCTGGTAGC
GGTGGTTTTTTTGTTTGCAAGCAGCAGATTACGCGCAGAAAAAAAGGATCTCAAGAAGATCCTTTGATCTTTTCTACGGGGTCTGACGCT
CAGTGGAACGAAAACTCACGTTAAGGGATTTTGGTCATGAGATTATCAAAAAGGATCTTCACCTAGATCCTTTTAAATTAAAAATGAAG
TTTTAAATCAATCTAAAGTATATATGAGTAAACTTGGTCTGACAGTTACCAATGCTTAATCAGTGAGGCACCTATCTCAGCGATCTGTCT
ATTTCGTTCATCCATAGTTGCCTGACTCCCCGTCGTGTAGATAACTACGATACGGGAGGGCTTACCATCTGGCCCCAGTGCTGCAATGAT
ACCGCGAGACCCACGCTCACCGGCTCCAGATTTATCAGCAATAAACCAGCCAGCCGGAAGGGCCGAGCGCAGAAGTGGTCCTGCAACT
TTATCCGCCTCCATCCAGTCTATTAATTGTTGCCGGGAAGCTAGAGTAAGTAGTTCGCCAGTTAATAGTTTGCGCAACGTTGTTGCCATT
GCTACAGGCATCGTGGTGTCACGCTCGTCGTTTGGTATGGCTTCATTCAGCTCCGGTTCCCAACGATCAAGGCGAGTTACATGATCCCCC
ATGTTGTGCAAAAAAGCGGTTAGCTCCTTCGGTCCTCCGATCGTTGTCAGAAGTAAGTTGGCCGCAGTGTTATCACTCATGGTTATGGCA
GCACTGCATAATTCTCTTACTGTCATGCCATCCGTAAGATGCTTTTCTGTGACTGGTGAGTACTCAACCAAGTCATTCTGAGAATAGTGT
ATGCGGCGACCGAGTTGCTCTTGCCCGGCGTCAATACGGGATAATACCGCGCCACATAGCAGAACTTTAAAAGTGCTCATCATTGGAAA
ACGTTCTTCGGGGCGAAAACTCTCAAGGATCTTACCGCTGTTGAGATCCAGTTCGATGTAACCCACTCGTGCACCCAACTGATCTTCAGC
ATCTTTTACTTTCACCAGCGTTTCTGGGTGAGCAAAAACAGGAAGGCAAAATGCCGCAAAAAAGGGAATAAGGGCGACACGGAAATGT
TGAATACTCAT

PB-pCAG-PEmax-P2A-hMLH1dn-T2A-mcherry-BSD vector

Plasmid backbone

CAG promoter

SV40 NLS

SpCas9 (R221K N394K H840A)

c-myc NLS

T2A-mCherry

EF-1-alpha core promoter

BSD

ACTCTTCCTTTTTCAATATTATTGAAGCATTTATCAGGGTTATTGTCTCATGAGCGGATACATATTTGAATGTATTTAGAAAAATAAACA
AATAGGGGTTCCGCGCACATTTCCCCGAAAAGTGCCACCTAAATTGTAAGCGTTAATATTTTGTTAAAATTCGCGTTAAATTTTTGTTAA
ATCAGCTCATTTTTTAACCAATAGGCCGAAATCGGCAAAATCCCTTATAAATCAAAAGAATAGACCGAGATAGGGTTGAGTGTTGTTCC
AGTTTGGAACAAGAGTCCACTATTAAAGAACGTGGACTCCAACGTCAAAGGGCGAAAAACCGTCTATCAGGGCGATGGCCCACTACGT
GAACCATCACCCTAATCAAGTTTTTTGGGGTCGAGGTGCCGTAAAGCACTAAATCGGAACCCTAAAGGGAGCCCCCGATTTAGAGCTTG
ACGGGGAAAGCCGGCGAACGTGGCGAGAAAGGAAGGGAAGAAAGCGAAAGGAGCGGGCGCTAGGGCGCTGGCAAGTGTAGCGGTCA
CGCTGCGCGTAACCACCACACCCGCCGCGCTTAATGCGCCGCTACAGGGCGCGTCCCATTCGCCATTCAGGCTGCGCAACTGTTGGGAA
GGGCGATCGGTGCGGGCCTCTTCGCTATTACGCCAGCTGGCGAAAGGGGGATGTGCTGCAAGGCGATTAAGTTGGGTAACGCCAGGGT
TTTCCCAGTCACGACGTTGTAAAACGACGGCCAGTGAGCGCGCCTCGTTCATTCACGTTTTTGAACCCGTGGAGGACGGGCAGACTCGC
GGTGCAAATGTGTTTTACAGCGTGATGGAGCAGATGAAGATGCTCGACACGCTGCAGAACACGCAGCTAGATTAACCCTAGAAAGATA
ATCATATTGTGACGTACGTTAAAGATAATCATGTGTAAAATTGACGCATGTGTTTTATCGGTCTGTATATCGAGGTTTATTTATTAATTTG
AATAGATATTAAGTTTTATTATATTTACACTTACATACTAATAATAAATTCAACAAACAATTTATTTATGTTTATTTATTTATTAAAAAAA
ACAAAAACTCAAAATTTCTTCTATAAAGTAACAAAACTTTTATGAGGGACAGCCCCCCCCCAAAGCCCCCAGGGATGTAATTACGTCCC
TCCCCCGCTAGGGGGCAGCAGCGAGCCGCCCGGGGCTCCGCTCCGGTCCGGCGCTCCCCCCGCATCCCCGAGCCGGCAGCGTGCGGGG
ACAGCCCGGGCACGGGGAAGGTGGCACGGGATCGCTTTCCTCTGAACGCTTCTCGCTGCTCTTTGAGCCTGCAGACACCTGGGGGGATA
CGGGGAAAAGGCCTCCACGGCCGACATTGATTATTGACTAGTTATTAATAGTAATCAATTACGGGGTCATTAGTTCATAGCCCATATAT
GGAGTTCCGCGTTACATAACTTACGGTAAATGGCCCGCCTGGCTGACCGCCCAACGACCCCCGCCCATTGACGTCAATAATGACGTATG
TTCCCATAGTAACGCCAATAGGGACTTTCCATTGACGTCAATGGGTGGAGTATTTACGGTAAACTGCCCACTTGGCAGTACATCAAGTG
TATCATATGCCAAGTACGCCCCCTATTGACGTCAATGACGGTAAATGGCCCGCCTGGCATTATGCCCAGTACATGACCTTATGGGACTTT
CCTACTTGGCAGTACATCTACGTATTAGTCATCGCTATTACCATGGTCGAGGTGAGCCCCACGTTCTGCTTCACTCTCCCCATCTCCCCCC
CCTCCCCACCCCCAATTTTGTATTTATTTATTTTTTAATTATTTTGTGCAGCGATGGGGGCGGGGGGGGGGGGGGCGCGCGCCAGGCGGG

GCGGGGCGGGGCGAGGGGCGGGGCGGGGCGAGGCGGAGAGGTGCGGCGGCAGCCAATCAGAGCGGCGCGCTCCGAAAGTTTCCTTTT
ATGGCGAGGCGGCGGCGGCGGCGGCCCTATAAAAAGCGAAGCGCGCGGCGGGCGGGAGTCGCTGCGCGCTGCCTTCGCCCCGTGCCCC
GCTCCGCCGCCGCCTCGCGCCGCCCGCCCCGGCTCTGACTGACCGCGTTACTCCCACAGGTGAGCGGGCGGGACGGCCCTTCTCCTCCG
GGCTGTAATTAGCGCTTGGTTTAATGACGGCTTGTTTCTTTTCTGTGGCTGCGTGAAAGCCTTGAGGGGCTCCGGGAGGGCCCTTTGTGC
GGGGGGAGCGGCTCGGGGGGTGCGTGCGTGTGTGTGTGCGTGGGGAGCGCCGCGTGCGGCTCCGCGCTGCCCGGCGGCTGTGAGCGCT
GCGGGCGCGGCGCGGGGCTTTGTGCGCTCCGCAGTGTGCGCGAGGGGAGCGCGGCCGGGGGCGGTGCCCCGCGGTGCGGGGGGGGCTG
CGAGGGGAACAAAGGCTGCGTGCGGGGTGTGTGCGTGGGGGGGTGAGCAGGGGGTGTGGGCGCGTCGGTCGGGCTGCAACCCCCCCTG
CACCCCCCTCCCCGAGTTGCTGAGCACGGCCCGGCTTCGGGTGCGGGGCTCCGTACGGGGCGTGGCGCGGGGCTCGCCGTGCCGGGCGG
GGGGTGGCGGCAGGTGGGGGTGCCGGGCGGGGCGGGGCCGCCTCGGGCCGGGGAGGGCTCGGGGGAGGGGCGCGGCGGCCCCCGGAG
CGCCGGCGGCTGTCGAGGCGCGGCGAGCCGCAGCCATTGCCTTTTATGGTAATCGTGCGAGAGGGCGCAGGGACTTCCTTTGTCCCAAA
TCTGTGCGGAGCCGAAATCTGGGAGGCGCCGCCGCACCCCCTCTAGCGGGCGCGGGGCGAAGCGGTGCGGCGCCGGCAGGAAGGAAAT
GGGCGGGGAGGGCCTTCGTGCGTCGCCGCGCCGCCGTCCCCTTCTCCCTCTCCAGCCTCGGGGCTGTCCGCGGGGGGACGGCTGCCTTC
GGGGGGGACGGGGCAGGGCGGGGTTCGGCTTCTGGCGTGTGACCGGCGGCTCTAGAGCCTCTGCTAACCATGTTCATGCCTTCTTCTTTT
TCCTACAGCTCCTGGGCAACGTGCTGGTTATTGTGCTGTCTCATCATTTTGGCAAAGAATTCTAATACGACTCACTATAGGGAGAGCCGC
CACCATGAAACGGACAGCCGACGGAAGCGAGTTCGAGTCACCAAAGAAGAAGCGGAAAGTCGACAAGAAGTACAGCATCGGCCTGGA
CATCGGCACCAACTCTGTGGGCTGGGCCGTGATCACCGACGAGTACAAGGTGCCCAGCAAGAAATTCAAGGTGCTGGGCAACACCGAC
CGGCACAGCATCAAGAAGAACCTGATCGGAGCCCTGCTGTTCGACAGCGGCGAAACAGCCGAGGCCACCCGGCTGAAGAGAACCGCCA
GAAGAAGATACACCAGACGGAAGAACCGGATCTGCTATCTGCAAGAGATCTTCAGCAACGAGATGGCCAAGGTGGACGACAGCTTCTT
CCACAGACTGGAAGAGTCCTTCCTGGTGGAAGAGGATAAGAAGCACGAGCGGCACCCCATCTTCGGCAACATCGTGGACGAGGTGGCC
TACCACGAGAAGTACCCCACCATCTACCACCTGAGAAAGAAACTGGTGGACAGCACCGACAAGGCCGACCTGCGGCTGATCTATCTGG
CCCTGGCCCACATGATCAAGTTCCGGGGCCACTTCCTGATCGAGGGCGACCTGAACCCCGACAACAGCGACGTGGACAAGCTGTTCATC
CAGCTGGTGCAGACCTACAACCAGCTGTTCGAGGAAAACCCCATCAACGCCAGCGGCGTGGACGCCAAGGCCATCCTGTCTGCCAGAC
TGAGCAAGAGCAGAAAGCTGGAAAATCTGATCGCCCAGCTGCCCGGCGAGAAGAAGAATGGCCTGTTCGGAAACCTGATTGCCCTGAG
CCTGGGCCTGACCCCCAACTTCAAGAGCAACTTCGACCTGGCCGAGGATGCCAAACTGCAGCTGAGCAAGGACACCTACGACGACGAC
CTGGACAACCTGCTGGCCCAGATCGGCGACCAGTACGCCGACCTGTTTCTGGCCGCCAAGAACCTGTCCGACGCCATCCTGCTGAGCGA
CATCCTGAGAGTGAACACCGAGATCACCAAGGCCCCCCTGAGCGCCTCTATGATCAAGAGATACGACGAGCACCACCAGGACCTGACC
CTGCTGAAAGCTCTCGTGCGGCAGCAGCTGCCTGAGAAGTACAAAGAGATTTTCTTCGACCAGAGCAAGAACGGCTACGCCGGCTACAT
TGACGGCGGAGCCAGCCAGGAAGAGTTCTACAAGTTCATCAAGCCCATCCTGGAAAAGATGGACGGCACCGAGGAACTGCTCGTGAAG
CTGAAGAGAGAGGACCTGCTGCGGAAGCAGCGGACCTTCGACAACGGCAGCATCCCCCACCAGATCCACCTGGGAGAGCTGCACGCCA
TTCTGCGGCGGCAGGAAGATTTTTACCCATTCCTGAAGGACAACCGGGAAAAGATCGAGAAGATCCTGACCTTCCGCATCCCCTACTAC
GTGGGCCCTCTGGCCAGGGGAAACAGCAGATTCGCCTGGATGACCAGAAAGAGCGAGGAAACCATCACCCCCTGGAACTTCGAGGAAG
TGGTGGACAAGGGCGCTTCCGCCCAGAGCTTCATCGAGCGGATGACCAACTTCGATAAGAACCTGCCCAACGAGAAGGTGCTGCCCAA
GCACAGCCTGCTGTACGAGTACTTCACCGTGTATAACGAGCTGACCAAAGTGAAATACGTGACCGAGGGAATGAGAAAGCCCGCCTTC
CTGAGCGGCGAGCAGAAAAAGGCCATCGTGGACCTGCTGTTCAAGACCAACCGGAAAGTGACCGTGAAGCAGCTGAAAGAGGACTACT
TCAAGAAAATCGAGTGCTTCGACTCCGTGGAAATCTCCGGCGTGGAAGATCGGTTCAACGCCTCCCTGGGCACATACCACGATCTGCTG
AAAATTATCAAGGACAAGGACTTCCTGGACAATGAGGAAAACGAGGACATTCTGGAAGATATCGTGCTGACCCTGACACTGTTTGAGG
ACAGAGAGATGATCGAGGAACGGCTGAAAACCTATGCCCACCTGTTCGACGACAAAGTGATGAAGCAGCTGAAGCGGCGGAGATACA
CCGGCTGGGGCAGGCTGAGCCGGAAGCTGATCAACGGCATCCGGGACAAGCAGTCCGGCAAGACAATCCTGGATTTCCTGAAGTCCGA
CGGCTTCGCCAACAGAAACTTCATGCAGCTGATCCACGACGACAGCCTGACCTTTAAAGAGGACATCCAGAAAGCCCAGGTGTCCGGC
CAGGGCGATAGCCTGCACGAGCACATTGCCAATCTGGCCGGCAGCCCCGCCATTAAGAAGGGCATCCTGCAGACAGTGAAGGTGGTGG
ACGAGCTCGTGAAAGTGATGGGCCGGCACAAGCCCGAGAACATCGTGATCGAAATGGCCAGAGAGAACCAGACCACCCAGAAGGGAC
AGAAGAACAGCCGCGAGAGAATGAAGCGGATCGAAGAGGGCATCAAAGAGCTGGGCAGCCAGATCCTGAAAGAACACCCCGTGGAAA
ACACCCAGCTGCAGAACGAGAAGCTGTACCTGTACTACCTGCAGAATGGGCGGGATATGTACGTGGACCAGGAACTGGACATCAACCG
GCTGTCCGACTACGATGTGGACGCTATCGTGCCTCAGAGCTTTCTGAAGGACGACTCCATCGACAACAAGGTGCTGACCAGAAGCGACA
AGAACCGGGGCAAGAGCGACAACGTGCCCTCCGAAGAGGTCGTGAAGAAGATGAAGAACTACTGGCGGCAGCTGCTGAACGCCAAGC
TGATTACCCAGAGAAAGTTCGACAATCTGACCAAGGCCGAGAGAGGCGGCCTGAGCGAACTGGATAAGGCCGGCTTCATCAAGAGACA
GCTGGTGGAAACCCGGCAGATCACAAAGCACGTGGCACAGATCCTGGACTCCCGGATGAACACTAAGTACGACGAGAATGACAAGCTG
ATCCGGGAAGTGAAAGTGATCACCCTGAAGTCCAAGCTGGTGTCCGATTTCCGGAAGGATTTCCAGTTTTACAAAGTGCGCGAGATCAA
CAACTACCACCACGCCCACGACGCCTACCTGAACGCCGTCGTGGGAACCGCCCTGATCAAAAAGTACCCTAAGCTGGAAAGCGAGTTC
GTGTACGGCGACTACAAGGTGTACGACGTGCGGAAGATGATCGCCAAGAGCGAGCAGGAAATCGGCAAGGCTACCGCCAAGTACTTCT
TCTACAGCAACATCATGAACTTTTTCAAGACCGAGATTACCCTGGCCAACGGCGAGATCCGGAAGCGGCCTCTGATCGAGACAAACGGC
GAAACCGGGGAGATCGTGTGGGATAAGGGCCGGGATTTTGCCACCGTGCGGAAAGTGCTGAGCATGCCCCAAGTGAATATCGTGAAAA
AGACCGAGGTGCAGACAGGCGGCTTCAGCAAAGAGTCTATCCTGCCCAAGAGGAACAGCGATAAGCTGATCGCCAGAAAGAAGGACT
GGGACCCTAAGAAGTACGGCGGCTTCGACAGCCCCACCGTGGCCTATTCTGTGCTGGTGGTGGCCAAAGTGGAAAAGGGCAAGTCCAA
GAAACTGAAGAGTGTGAAAGAGCTGCTGGGGATCACCATCATGGAAAGAAGCAGCTTCGAGAAGAATCCCATCGACTTTCTGGAAGCC
AAGGGCTACAAAGAAGTGAAAAAGGACCTGATCATCAAGCTGCCTAAGTACTCCCTGTTCGAGCTGGAAAACGGCCGGAAGAGAATGC
TGGCCTCTGCCGGCGAACTGCAGAAGGGAAACGAACTGGCCCTGCCCTCCAAATATGTGAACTTCCTGTACCTGGCCAGCCACTATGAG
AAGCTGAAGGGCTCCCCCGAGGATAATGAGCAGAAACAGCTGTTTGTGGAACAGCACAAGCACTACCTGGACGAGATCATCGAGCAGA
TCAGCGAGTTCTCCAAGAGAGTGATCCTGGCCGACGCTAATCTGGACAAAGTGCTGTCCGCCTACAACAAGCACCGGGATAAGCCCATC
AGAGAGCAGGCCGAGAATATCATCCACCTGTTTACCCTGACCAATCTGGGAGCCCCTGCCGCCTTCAAGTACTTTGACACCACCATCGA
CCGGAAGAGGTACACCAGCACCAAAGAGGTGCTGGACGCCACCCTGATCCACCAGAGCATCACCGGCCTGTACGAGACACGGATCGAC
CTGTCTCAGCTGGGAGGTGACTCCGGCGGAAGCTCTGGTGGCAGCAAGCGGACCGCCGACGGCTCTGAATTCGAGAGCCCTAAGAAGA
AAAGAAAGGTGAGCGGAGGCTCTAGCGGCGGAAGCACCCTGAACATTGAAGACGAGTATAGACTGCATGAAACAAGCAAGGAACCCG
ACGTGTCCCTGGGCTCCACCTGGCTGTCCGACTTTCCCCAGGCCTGGGCCGAGACAGGAGGAATGGGCCTGGCCGTGCGGCAGGCACCC
CTGATCATCCCTCTGAAGGCCACCTCTACACCCGTGAGCATCAAGCAGTACCCTATGTCTCAGGAGGCCAGACTGGGCATCAAGCCTCA
CATCCAGAGGCTGCTGGACCAGGGCATCCTGGTGCCATGCCAGAGCCCCTGGAACACACCACTGCTGCCCGTGAAGAAGCCAGGCACC
AATGACTATAGACCCGTGCAGGATCTGAGAGAGGTGAACAAGAGGGTGGAGGATATCCACCCCACCGTGCCCAACCCTTACAATCTGC
TGTCCGGCCTGCCCCCTTCTCACCAGTGGTATACAGTGCTGGACCTGAAGGATGCCTTCTTTTGTCTGAGACTGCACCCTACCAGCCAGC
CACTGTTCGCCTTTGAGTGGAGGGACCCTGAGATGGGCATCTCTGGCCAGCTGACCTGGACACGCCTGCCTCAGGGCTTCAAGAATAGC
CCAACACTGTTTAACGAGGCCCTGCACCGCGACCTGGCAGATTTCCGGATCCAGCACCCAGATCTGATCCTGCTGCAGTACGTGGACGA
TCTGCTGCTGGCCGCCACCAGCGAGCTGGATTGCCAGCAGGGAACACGCGCCCTGCTGCAGACCCTGGGAAACCTGGGATATAGGGCA
TCCGCCAAGAAGGCCCAGATCTGTCAGAAGCAGGTGAAGTACCTGGGCTATCTGCTGAAGGAGGGCCAGAGATGGCTGACAGAGGCCA

GGAAGGAGACAGTGATGGGCCAGCCAACACCCAAGACCCCAAGACAGCTGAGGGAGTTCCTGGGCAAAGCAGGATTTTGCAGGCTGTT
CATCCCAGGATTCGCAGAGATGGCAGCACCTCTGTACCCACTGACCAAGCCGGGCACCCTGTTTAATTGGGGCCCTGACCAGCAGAAGG
CCTATCAGGAGATCAAGCAGGCCCTGCTGACAGCACCAGCCCTGGGCCTGCCAGACCTGACCAAGCCTTTCGAGCTGTTTGTGGATGAG
AAGCAGGGCTACGCCAAGGGCGTGCTGACCCAGAAGCTGGGACCATGGAGACGGCCCGTGGCCTATCTGTCCAAGAAGCTGGACCCAG
TGGCAGCAGGATGGCCACCATGCCTGAGGATGGTGGCAGCAATCGCCGTGCTGACAAAGGATGCCGGCAAGCTGACCATGGGACAGCC
ACTGGTCATCCTGGCACCACACGCAGTGGAGGCCCTGGTGAAGCAGCCTCCAGATCGCTGGCTGTCTAACGCCCGGATGACACACTACC
AGGCCCTGCTGCTGGACACCGATCGCGTGCAGTTTGGCCCTGTGGTGGCCCTGAATCCAGCCACCCTGCTGCCTCTGCCAGAGGAGGGC
CTGCAGCACAACTGTCTGGACATCCTGGCAGAGGCACACGGAACAAGGCCAGACCTGACCGATCAGCCCCTGCCTGACGCCGATCACA
CATGGTATACCGATGGAAGCTCCCTGCTGCAGGAGGGCCAGAGGAAGGCAGGAGCAGCAGTGACCACAGAGACAGAAGTGATCTGGG
CCAAGGCCCTGCCAGCAGGCACATCCGCCCAGCGGGCCGAGCTGATCGCCCTGACCCAGGCCCTGAAGATGGCCGAGGGCAAGAAGCT
GAACGTGTACACAGACTCCAGATATGCCTTCGCCACCGCACACATCCACGGAGAGATCTACAGGCGCCGGGGCTGGCTGACCTCTGAG
GGCAAGGAGATCAAGAACAAGGATGAGATCCTGGCCCTGCTGAAGGCCCTGTTTCTGCCCAAGCGGCTGAGCATCATCCACTGTCCTGG
ACACCAGAAGGGACACTCCGCCGAGGCAAGGGGCAATCGGATGGCCGACCAGGCCGCCAGAAAGGCTGCTATTACTGAAACTCCCGAC
ACTTCCACTCTGCTGATTGAAAACTCCTCCCCTTCTGGCGGCTCAAAAAGAACCGCCGACGGCAGCGAATTCGAGTCTCCCAAGAAGAA
GAGGAAAGTCGGCTCTGGCCCTGCCGCTAAGAGAGTGAAGCTGGACGGAAGCGGAGCTACTAACTTCAGCCTGCTGAAGCAGGCTGGA
GACGTGGAGGAGAACCCTGGACCTAGCTTCGTTGCTGGAGTCATCCGGAGACTGGACGAGACAGTGGTGAACAGAATTGCCGCCGGCG
AGGTGATCCAGAGACCTGCCAATGCAATAAAGGAGATGATCGAGAACTGTCTGGACGCCAAGTCCACAAGCATTCAGGTGATCGTGAA
GGAGGGCGGACTGAAGCTGATCCAGATCCAAGACAACGGCACAGGCATCAGAAAGGAAGATCTGGACATCGTGTGTGAACGGTTCACC
ACATCTAAGCTGCAGTCTTTTGAGGATCTGGCCTCTATCAGTACCTACGGCTTCAGAGGCGAGGCCCTGGCCAGCATCAGCCACGTGGC
CCATGTGACCATCACCACCAAAACCGCCGACGGCAAATGCGCTTATCGCGCTAGCTACAGCGACGGCAAGCTGAAAGCCCCGCCAAAG
CCTTGCGCCGGCAACCAGGGTACACAGATAACAGTGGAGGATCTGTTCTACAACATCGCCACCCGGAGAAAGGCCCTGAAAAATCCCA
GCGAGGAGTACGGCAAGATCCTGGAAGTCGTCGGCAGATACTCCGTGCACAACGCCGGAATCAGCTTTAGCGTAAAGAAGCAGGGAGA
AACCGTGGCCGATGTGCGCACCCTGCCAAATGCCAGCACCGTGGATAACATCAGAAGCATTTTCGGAAATGCCGTGTCCAGAGAACTG
ATCGAGATCGGCTGCGAAGATAAGACCCTGGCTTTTAAGATGAACGGCTACATCAGCAACGCCAATTACTCTGTGAAGAAGTGCATCTT
TCTTCTGTTCATCAACCACAGACTGGTGGAAAGCACCAGCCTGCGGAAAGCCATCGAGACAGTGTACGCCGCCTACCTGCCTAAGAACA
CCCACCCCTTCCTGTACCTGAGCCTCGAGATCAGCCCTCAGAACGTGGACGTCAATGTGCATCCTACAAAGCACGAGGTGCACTTCCTG
CACGAGGAAAGCATCCTGGAAAGAGTGCAGCAGCACATTGAGAGCAAGCTGCTGGGCTCTAACAGCAGCAGAATGTACTTCACACAGA
CCCTGCTGCCTGGCCTGGCCGGCCCCTCAGGCGAAATGGTTAAGTCCACAACCTCTCTGACCTCATCCAGCACCAGCGGTTCTTCCGATA
AGGTGTACGCCCACCAGATGGTGCGGACCGACTCTCGGGAGCAGAAGCTGGACGCCTTTCTGCAACCTCTGAGCAAACCTCTGAGCTCT
CAGCCTCAGGCCATCGTGACCGAGGACAAGACAGATATCTCCTCCGGCCGTGCCAGACAGCAGGACGAAGAAATGCTCGAGCTGCCAG
CTCCTGCCGAGGTGGCCGCCAAGAACCAGAGCCTGGAGGGAGATACCACAAAGGGCACCAGCGAAATGAGCGAGAAGCGGGGCCCTA
CCTCCAGCAACCCCAGAAAACGGCACCGGGAGGACAGCGACGTGGAAATGGTGGAGGACGACAGCAGAAAGGAAATGACAGCCGCTT
GTACCCCTAGAAGAAGAATCATCAACCTGACCTCCGTGCTGAGCCTGCAGGAGGAGATCAACGAGCAGGGCCACGAGGTGCTGAGAGA
GATGCTGCACAATCACAGCTTCGTGGGCTGCGTGAACCCTCAATGGGCCCTGGCTCAGCATCAAACAAAGCTGTACCTGCTGAACACCA
CCAAGCTGAGCGAAGAGCTGTTCTACCAGATCCTCATCTACGACTTCGCCAACTTCGGCGTGCTACGCCTGAGCGAGCCCGCCCCTCTGT
TTGACCTGGCCATGCTGGCTCTGGATAGCCCAGAAAGCGGCTGGACAGAAGAGGACGGACCTAAAGAGGGGCTGGCTGAATACATCGT
GGAGTTCCTGAAGAAAAAGGCCGAGATGCTGGCCGACTACTTTTCTCTGGAAATCGACGAGGAAGGCAACCTGATCGGCCTGCCTCTGC
TGATCGATAACTACGTGCCTCCCCTGGAAGGCCTGCCCATCTTCATCCTGAGACTGGCTACAGAGGTGAACTGGGACGAGGAAAAGGA
ATGCTTCGAGTCTCTGAGCAAGGAGTGCGCCATGTTCTATAGCATCAGAAAACAGTACATCTCTGAAGAGAGCACTCTGTCTGGCCAGC
AGAGTGAAGTGCCCGGAAGCATCCCCAACAGCTGGAAGTGGACCGTGGAACACATCGTGTACAAGGCCCTGCGGAGCCACATTCTCCC
TCCTAAGCACTTCACCGAGGACGGCAACATCCTGCAGCTGGCCAACCTGCCCGACCTTTATAAGGTTTTCGGCAGTGGAGAGGGCAGAG
GAAGTCTGCTAACATGCGGTGACGTCGAGGAGAATCCTGGCCCAATGGTGAGCAAGGGCGAGGAGGATAACATGGCCATCATCAAGGA
GTTCATGCGCTTCAAGGTGCACATGGAGGGCTCCGTGAACGGCCACGAGTTCGAGATCGAGGGCGAGGGCGAGGGCCGCCCCTACGAG
GGCACCCAGACCGCCAAGCTGAAGGTGACCAAGGGTGGCCCCCTGCCCTTCGCCTGGGACATCCTGTCCCCTCAGTTCATGTACGGCTC
CAAGGCCTACGTGAAGCACCCCGCCGACATCCCCGACTACTTGAAGCTGTCCTTCCCCGAGGGCTTCAAGTGGGAGCGCGTGATGAACT
TCGAGGACGGCGGCGTGGTGACCGTGACCCAGGACTCCTCCCTGCAGGACGGCGAGTTCATCTACAAGGTGAAGCTGCGCGGCACCAA
CTTCCCCTCCGACGGCCCCGTAATGCAGAAGAAGACCATGGGCTGGGAGGCCTCCTCCGAGCGGATGTACCCCGAGGACGGCGCCCTG
AAGGGCGAGATCAAGCAGAGGCTGAAGCTGAAGGACGGCGGCCACTACGACGCTGAGGTCAAGACCACCTACAAGGCCAAGAAGCCC
GTGCAGCTGCCCGGCGCCTACAACGTCAACATCAAGTTGGACATCACCTCCCACAACGAGGACTACACCATCGTGGAACAGTACGAAC
GCGCCGAGGGCCGCCACTCCACCGGCGGCATGGACGAGCTGTACAAGTAAGTTTAAACGGGGGAGGCTAACTGAAACACGGAAGGAG
ACAATACCGGAAGGAACCCGCGCTATGACGGCAATAAAAAGACAGAATAAAACGCACGGGTGTTGGGTCGTTTGTTCATAAACGCGGG
GTTCGGTCCCAGGGCTGGCACTCTGTCGATACCCCACCGAGACCCCATTGGGGCCAATACGCCCGCGTTTCTTCCTTTTCCCCACCCCAC
CCCCCAAGTTCGGGTGAAGGCCCAGGGCTCGCAGCCAACGTCGGGGCGGCAGGCCCTGCCATAGCAGATCTGCGAAATCGGATCCGCG
GCCGCACTGATTCTAGCTGCAGAGGCTCCGGTGCCCGTCAGTGGGCAGAGCGCACATCGCCCACAGTCCCCGAGAAGTTGGGGGGAGG
GGTCGGCAATTGAACCGGTGCCTAGAGAAGGTGGCGCGGGGTAAACTGGGAAAGTGATGTCGTGTACTGGCTCCGCCTTTTTCCCGAGG
GTGGGGGAGAACCGTATATAAGTGCAGTAGTCGCCGTGAACGTTCTTTTTCGCAACGGGTTTGCCGCCAGAACACAGGATCCTCGAGCC
ACCATGGCCAAGCCTTTGTCTCAAGAAGAATCCACCCTCATTGAAAGAGCAACGGCTACAATCAACAGCATCCCCATCTCTGAAGACTA
CAGCGTCGCCAGCGCAGCTCTCTCTAGCGACGGCCGCATCTTCACTGGTGTCAATGTATATCATTTTACTGGGGGACCTTGTGCAGAACT
CGTGGTGCTGGGCACTGCTGCTGCTGCGGCAGCTGGCAACCTGACTTGTATCGTCGCGATCGGAAATGAGAACAGGGGCATCTTGAGCC
CCTGCGGACGGTGCCGACAGGTGCTTCTCGATCTGCATCCTGGGATCAAAGCCATAGTGAAGGACAGTGATGGACAGCCGACGGCAGT
TGGGATTCGTGAATTGCTGCCCTCTGGTTATGTGTGGGAGGGCTAAGTTAACTTGTTTATTGCAGCTTATAATGGTTACAAATAAAGCAA
TAGCATCACAAATTTCACAAATAAAGCATTTTTTTCACTGCATTCTAGTTGTGGTTTGTCCAAACTCATCAATGTATCTTATCATGTCTGG
AATTGACTCAAATGATGTCAATTAGTCTATCAGAAGCTCATCTGGTCTCCCTTCCGGGGGACAAGACATCCCTGTTTAATATTTAAACAG
CAGTGTTCCCAAACTGGGTTCTTATATCCCTTGCTCTGGTCAACCAGGTTGCAGGGTTTCCTGTCCTCACAGGAACGAAGTCCCTAAAGA
AACAGTGGCAGCCAGGTTTAGCCCCGGAATTGACTGGATTCCTTTTTTAGGGCCCATTGGTATGGCTTTTTCCCCGTATCCCCCCAGGTG
TCTGCAGGCTCAAAGAGCAGCGAGAAGCGTTCAGAGGAAAGCGATCCCGTGCCACCTTCCCCGTGCCCGGGCTGTCCCCGCACGCTGCC
GGCTCGGGGATGCGGGGGGAGCGCCGGACCGGAGCGGAGCCCCGGGCGGCTCGCTGCTGCCCCCTAGCGGGGGAGGGACGTAATTAC
ATCCCTGGGGGCTTTGGGGGGGGGCTGTCCCTGATATCTATAACAAGAAAATATATATATATAATAAGTTATCACGTAAGTAGAACATGAA
ATAACAATATAATTATCGTATGAGTTAAATCTTAAAAGTCACGTAAAAGATAATCATGCGTCATTTTGACTCACGCGGTCGTTATAGTTC
AAAATCAGTGACACTTACCGCATTGACAAGCACGCCTCACGGGAGCTCCAAGCGGCGACTGAGATGTCCTAAATGCACAGCGACGGAT
TCGCGCTATTTAGAAAGAGAGAGCAATATTTCAAGAATGCATGCGTCAATTTTACGCAGACTATCTTTCTAGGGTTAATCTAGCTGCATC

AGGATCATATCGTCGGGTCTTTTTTCCGGCTCAGTCATCGCCCAAGCTGGCGCTATCTGGGCATCGGGGAGGAAGAAGCCCGTGCCTTTT
CCCGCGAGGTTGAAGCGGCATGGAAAGAGTTTGCCGAGGATGACTGCTGCTGCATTGACGTTGAGCGAAAACGCACGTTTACCATGAT
GATTCGGGAAGGTGTGGCCATGCACGCCTTTAACGGTGAACTGTTCGTTCAGGCCACCTGGGATACCAGTTCGTCGCGGCTTTTCCGGA
CACAGTTCCGGATGGTCAGCCCGAAGCGCATCAGCAACCCGAACAATACCGGCGACAGCCGGAACTGCCGTGCCGGTGTGCAGATTAA
TGACAGCGGTGCGGCGCTGGGATATTACGTCAGCGAGGACGGGTATCCTGGCTGGATGCCGCAGAAATGGACATGGATACCCCGTGAG
TTACCCGGCGGGCGCGCTTGGCGTAATCATGGTCATAGCTGTTTCCTGTGTGAAATTGTTATCCGCTCACAATTCCACACAACATACGAG
CCGGAAGCATAAAGTGTAAAGCCTGGGGTGCCTAATGAGTGAGCTAACTCACATTAATTGCGTTGCGCTCACTGCCCGCTTTCCAGTCG
GGAAACCTGTCGTGCCAGCTGCATTAATGAATCGGCCAACGCGCGGGGAGAGGCGGTTTGCGTATTGGGCGCTCTTCCGCTTCCTCGCT
CACTGACTCGCTGCGCTCGGTCGTTCGGCTGCGGCGAGCGGTATCAGCTCACTCAAAGGCGGTAATACGGTTATCCACAGAATCAGGGG
ATAACGCAGGAAAGAACATGTGAGCAAAAGGCCAGCAAAAGGCCAGGAACCGTAAAAAGGCCGCGTTGCTGGCGTTTTTCCATAGGCT
CCGCCCCCCTGACGAGCATCACAAAAATCGACGCTCAAGTCAGAGGTGGCGAAACCCGACAGGACTATAAAGATACCAGGCGTTTCCC
CCTGGAAGCTCCCTCGTGCGCTCTCCTGTTCCGACCCTGCCGCTTACCGGATACCTGTCCGCCTTTCTCCCTTCGGGAAGCGTGGCGCTTT
CTCATAGCTCACGCTGTAGGTATCTCAGTTCGGTGTAGGTCGTTCGCTCCAAGCTGGGCTGTGTGCACGAACCCCCCGTTCAGCCCGACC
GCTGCGCCTTATCCGGTAACTATCGTCTTGAGTCCAACCCGGTAAGACACGACTTATCGCCACTGGCAGCAGCCACTGGTAACAGGATT
AGCAGAGCGAGGTATGTAGGCGGTGCTACAGAGTTCTTGAAGTGGTGGCCTAACTACGGCTACACTAGAAGAACAGTATTTGGTATCTG
CGCTCTGCTGAAGCCAGTTACCTTCGGAAAAAGAGTTGGTAGCTCTTGATCCGGCAAACAAACCACCGCTGGTAGCGGTGGTTTTTTTGT
TTGCAAGCAGCAGATTACGCGCAGAAAAAAAGGATCTCAAGAAGATCCTTTGATCTTTTCTACGGGGTCTGACGCTCAGTGGAACGAAA
ACTCACGTTAAGGGATTTTGGTCATGAGATTATCAAAAAGGATCTTCACCTAGATCCTTTTAAATTAAAAATGAAGTTTTAAATCAATCT
AAAGTATATATGAGTAAACTTGGTCTGACAGTTACCAATGCTTAATCAGTGAGGCACCTATCTCAGCGATCTGTCTATTTCGTTCATCCA
TAGTTGCCTGACTCCCCGTCGTGTAGATAACTACGATACGGGAGGGCTTACCATCTGGCCCCAGTGCTGCAATGATACCGCGAGACCCA
CGCTCACCGGCTCCAGATTTATCAGCAATAAACCAGCCAGCCGGAAGGGCCGAGCGCAGAAGTGGTCCTGCAACTTTATCCGCCTCCAT
CCAGTCTATTAATTGTTGCCGGGAAGCTAGAGTAAGTAGTTCGCCAGTTAATAGTTTGCGCAACGTTGTTGCCATTGCTACAGGCATCGT
GGTGTCACGCTCGTCGTTTGGTATGGCTTCATTCAGCTCCGGTTCCCAACGATCAAGGCGAGTTACATGATCCCCCATGTTGTGCAAAAA
AGCGGTTAGCTCCTTCGGTCCTCCGATCGTTGTCAGAAGTAAGTTGGCCGCAGTGTTATCACTCATGGTTATGGCAGCACTGCATAATTC
TCTTACTGTCATGCCATCCGTAAGATGCTTTTCTGTGACTGGTGAGTACTCAACCAAGTCATTCTGAGAATAGTGTATGCGGCGACCGAG
TTGCTCTTGCCCGGCGTCAATACGGGATAATACCGCGCCACATAGCAGAACTTTAAAAGTGCTCATCATTGGAAAACGTTCTTCGGGGC
GAAAACTCTCAAGGATCTTACCGCTGTTGAGATCCAGTTCGATGTAACCCACTCGTGCACCCAACTGATCTTCAGCATCTTTTACTTTCA
CCAGCGTTTCTGGGTGAGCAAAAACAGGAAGGCAAAATGCCGCAAAAAAGGGAATAAGGGCGACACGGAAATGTTGAATACTCAT
